# Mechanistic studies on single-electron transfer in frustrated Lewis pairs and its application to main-group chemistry†

**DOI:** 10.1039/d4cs00185k

**Published:** 2024-04-16

**Authors:** Lars J. C. van der Zee, Jelle Hofman, Joost M. van Gaalen, J. Chris Slootweg

**Affiliations:** a Van 't Hoff Institute for Molecular Sciences, University of Amsterdam PO box 94157 1090 GD Amsterdam The Netherlands j.c.slootweg@uva.nl

## Abstract

Advances in the field of frustrated Lewis pair (FLP) chemistry have led to the discovery of radical pairs, obtained by a single-electron transfer (SET) from the Lewis base to the Lewis acid. Radical pairs are intriguing for their potential to enable cooperative activation of challenging substrates (*e.g.*, CH_4_, N_2_) in a homolytic fashion, as well as the exploration of novel radical reactions. In this review, we will cover the two known mechanisms of SET in FLPs—thermal and photoinduced—along with methods (*i.e.*, CV, DFT, UV-vis) to predict the mechanism and to characterise the involved electron donors and acceptors. Furthermore, the available techniques (*i.e.*, EPR, UV-vis, transient absorption spectroscopy) for studying the corresponding radical pairs will be discussed. Initially, two model systems (PMes_3_/CPh_3_^+^ and PMes_3_/B(C_6_F_5_)_3_) will be reviewed to highlight the difference between a thermal and a photoinduced SET mechanism. Additionally, three cases are analysed to provide further tools and insights into characterizing electron donors and acceptors, and the associated radical pairs. Firstly, a thermal SET process between LiHMDS and [TEMPO][BF_4_] is discussed. Next, the influence of Lewis acid complexation on the electron acceptor will be highlighted to facilitate a SET between (*p*BrPh)_3_N and TCNQ. Finally, an analysis of sulfonium salts as electron acceptors will demonstrate how to manage systems with rapidly decomposing radical species. This framework equips the reader with an expanded array of tools for both predicting and characterizing SET events within FLP chemistry, thereby enabling its extension and application to the broader domain of main-group (photo)redox chemistry.

## Abstract 2

Here, we discuss predicting thermal and photoinduced single-electron transfer (SET) in frustrated Lewis pairs (FLPs) and characterizing the resulting radical pair. We then extend these methods' application to main-group chemistry.

## Graphical abstract

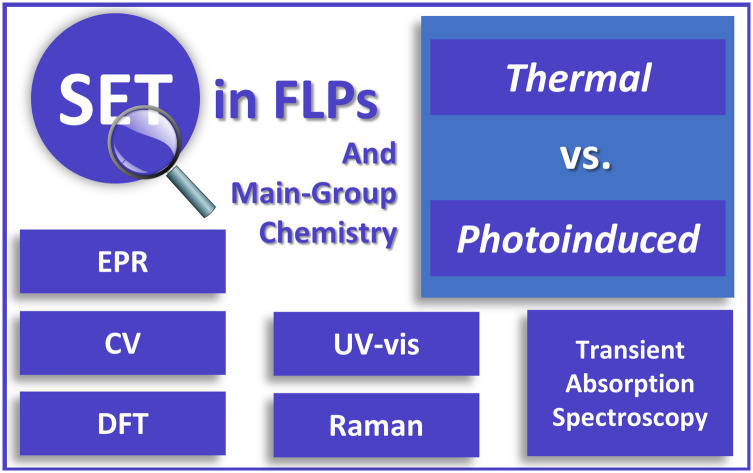

**Key learning points**

(1) How to predict the feasibility of both photoinduced and thermal SET in frustrated Lewis pairs and main-group chemistry based on redox potentials as well as ionisation energies and electron affinities.

(2) Characterisation of the ground-state charge transfer or electron donor–acceptor (EDA) complex for a photoinduced SET.

(3) How to distinguish experimentally between thermal and photoinduced SET in main-group (photo)redox chemistry.

(4) The available spectroscopic methods for characterising the formed radical pair, with regards to the specific conditions for both photoinduced and thermal SET.

(5) The limitations of the available spectroscopic methods concerning reactive radical pairs.

## 1. Introduction

**Scheme 1:**
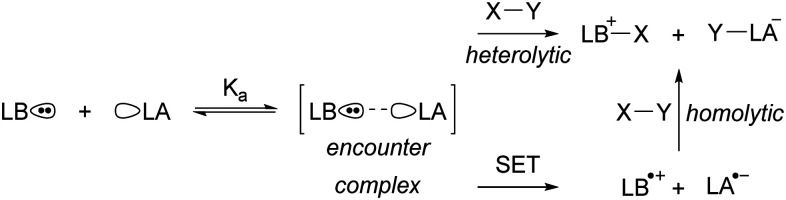
The equilibrium between a Lewis base (LB) and Lewis acid (LA) and the encounter complex with equilibrium constant *K*_a_. Subsequently, the synergistic, closed-shell activation of substrate X–Y can occur or potentially single electron transfer (SET) to afford a radical pair that can homolytically cleave X–Y.

Since the seminal report on frustrated Lewis pairs (FLPs) in 2006 by the group of Stephan,^1,2^ the field of sterically encumbered electron donor–acceptor pairs in main-group chemistry has centred around the heterolytic activation of substrates.^3–5^ Over the years, numerous substrates, including dihydrogen and carbon dioxide, have been successfully activated in this manner, and the reaction mechanisms have been elucidated, both experimentally and computationally.^6^ Generally, the chemistry begins with the formation of the so-called encounter complex (Scheme 1) before activation of the substrate through the cooperative action of the lone pair of the Lewis base and the vacant orbital of the Lewis acid. Despite the tremendous increase in the number of substrates that can be activated, the activation of rather inert and difficult-to-polarise substrates, such as methane or dinitrogen, still remains an unresolved challenge in main group chemistry.

**Scheme 2:**
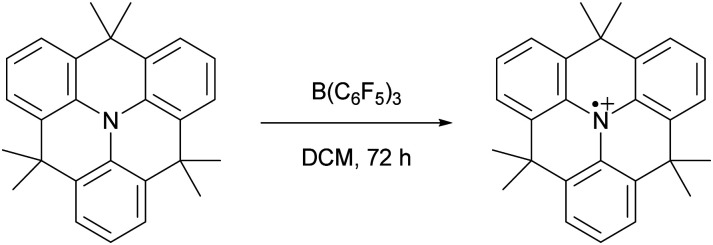
The first reported example of an SET between B(C_6_F_5_)_3_ and an organic electron donor, in this case a triarylamine, as reported by Wang *et al*.

In 2013, Wang and colleagues described the single-electron oxidation of a triarylamine by B(C_6_F_5_)_3_, demonstrating the potential for generating radicals in FLP systems (Scheme 2), although only the amine radical cation was characterised.^7^ This groundbreaking discovery opens the door for homolytic bond activation reactions through the cooperative action of both radicals, ideally augmenting the established closed-shell reactivity and paving the way for innovative radical reaction pathways of substrates like methane and dinitrogen. Since Wang *et al.*'s initial discovery, the variety of radicals reported in FLP chemistry has been on the rise.^8–10^ For instance, the detection of the PMes_3_˙^+^ radical cation in solutions containing PMes_3_/B(C_6_F_5_)_3_ and PMes_3_/Al(C_6_F_5_)_3_ by Stephan and colleagues has been key for the progress in the field of radical chemistry in FLPs.^11^ Moreover, Klare, Müller and colleagues have demonstrated the occurrence of a SET transfer in FLP systems, for example, through the oxidation of PMes_3_ by the trityl cation (CPh_3_^+^).^12^

**Scheme 3:**
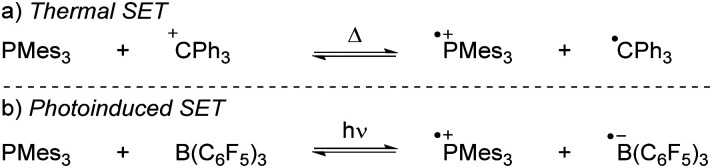
The thermally and photoinduced formed radical pairs of the model systems discussed in the Sections 2 and 3 in this tutorial review.

More recently, our group has focused on the mechanism of radical formation in FLP systems and demonstrated how a single electron transfer (SET) from a Lewis base to a Lewis acid could occur (Scheme 3).^13^ We identified two scenarios: the first being a thermal SET, where the electron is spontaneously transferred upon mixing the electron donor and acceptor. Intrigued by the work of Kochi,^14^ we explored the second scenario, a photoinduced SET, which aligns with Mulliken's theory of electron-donor acceptor (EDA) complexes.^15^ Alternatively, instead of forming an EDA complex, a radical pair could be formed by excitation of the electron donor or acceptor. Such an excited state, donor* or acceptor*, acts as a more potent electron donor or acceptor, respectively, and can form the corresponding radical pair through diffusional collision. While such pathways are well-established in photochemistry,^14^ they have not yet been reported in FLP chemistry and are therefore beyond the scope of this review.

In the EDA complex, or encounter complex, there exists an attractive interaction between the electron donor and acceptor, resulting in the emergence of a new absorption band, the so-called charge transfer (CT) band, which induces a SET upon irradiation.^14,15^ The wavelength of the CT-band can be determined using the following formula:*λ*_CT_ = IE + EA + *ω*Here, the IE represents the ionisation energy of the electron donor, EA is the electron affinity of the electron acceptor, and the electronic coupling term *ω* accounts for the interaction between the donor and the acceptor. This involves the mixing of the orbitals, typically the HOMO of the donor and LUMO of the acceptor, leading to changes in the orbital energies of the HOMO and LUMO of the CT-complex compared to the separate components. Therefore, the larger the interaction between the donor and acceptor, the more significant the *ω* term becomes in determining the excitation wavelength.

For both thermal and photoinduced SETs, a radical pair is obtained, which is typically in the form of a radical ion pair (RIP), consisting of the oxidised Lewis base as a radical cation and the reduced Lewis acid as the radical anion. In this tutorial review, we aim to discuss various methods for investigating SET processes in FLP chemistry and its application to the broader field of main group (photo)redox chemistry. Initially, we will consider the characterisation of individual electron donors and electron acceptors. This facilitates the prediction of the feasibility of thermal and/or photoinduced SET. Subsequently, we will explore methods suitable for characterising the formed radical pair, for both thermal and photoinduced SET. Moreover, the spectroscopic methods presented in this section can elucidate the actual mechanism of the SET. To guide the discussion, we will focus on two model systems: PMes_3_/CPh_3_^+^^12^ and PMes_3_/B(C_6_F_5_)_3_^13^ (Scheme 3). Switching the Lewis acid from the trityl cation to B(C_6_F_5_)_3_ alters the SET mechanism, as PMes_3_/CPh_3_^+^ undergoes a thermal SET, while PMes_3_/B(C_6_F_5_)_3_ experiences a photoinduced SET upon irradiation with visible light. Although these two model systems provide a framework for understanding photoinduced and thermal SETs in sterically encumbered donor–acceptor complexes, not all combinations of donors and acceptors will behave similarly. Therefore, we will also focus on three other, well-researched examples of SET in main-group chemistry. Firstly, the thermal SET between bis(trimethylsilyl)amide (HMDS^−^) and TEMPO^+^ is discussed, resulting in a radical pair capable of regioselective C–H activation of aliphatic substrates.^16^ Due to the limited stability of the formed HMDS˙, full characterisation of the radical pair could not be achieved by EPR spectroscopy. Therefore, a trapping experiment with styrene was performed to confirm the presence of both radicals, and additional mechanistic insights were obtained through radical clock and kinetic isotope effect (KIE) experiments. Secondly, the use of tetracyanoquinodimethane (TCNQ) as electron acceptor with (*p*BrPh)_3_N as the electron donor is discussed.^17^ The role of B(C_6_F_5_)_3_ as a coordinating Lewis acid to increase the electron affinity of TCNQ will be highlighted. Finally, the third example showcases photoinduced SET towards sulfonium salts, where the formed sulfonium radical undergoes rapid homolytic bond cleavage to form CF_3_˙, enabling the subsequent trifluoromethylation of substrates.^18^ Trapping of *in situ* generated CF_3_˙ with *N-tert*-butyl-α-phenylnitrone (PBN) results in a long-lived radical observable by EPR spectroscopy. This example demonstrates that for productive photochemistry the back-electron transfer (BET) should be outcompeted by a productive chemical transformation of one of the radicals.

## 2. Characterisation of the electron donor, electron acceptor, and EDA-complex

Each electron donor and electron acceptor can be characterised by its inherent redox properties before a SET transforms the combination into a radical pair. In this section, we will discuss the influence of the redox potentials of the individual components of the electron-donor acceptor pair on the mechanism of the SET and how to predict whether the SET process is thermal or photoinduced. This will be done both experimentally, using cyclic voltammetry (CV), and theoretically, using density functional theory (DFT) calculations. Furthermore, in case of a photoinduced SET, the closed-shell state (or ground state) consists of an EDA complex. Therefore, this section concludes with a brief discussion on how EDA complexes can be characterised by UV-vis spectroscopy.

### 2.1. Influence of redox potentials on the SET process

For a thermal SET to be observable by EPR spectroscopy, the radical pair should be less than approximately 0.4 eV higher in energy than the closed-shell state. According to the Boltzmann distribution, this condition yields a detectable concentration of radicals.^8,13^ Note, by definition, for a single electron transfer, 1.0 eV in DFT calculations equals 1.0 V in cyclic voltammetry (CV), which equals 23 kcal mol^−1^ difference in energy. The energy difference between the closed-shell state of the electron donor–acceptor pair and the radical pair state, Δ*E*_SET_, can be determined using the standard electrode potentials according to:Δ*E*_SET,CV_ = *E*_red_ − *E*_ox_Here *E*_red_ is the standard potential of the electron acceptor and *E*_ox_ is the standard potential of the electron donor.^19^ It is important to note that the convention used for determining Δ*E*_SET_ with electrochemistry differs in sign compared to conventions used for the *in silico* determination of the ionisation energies (IEs) and electron affinities (EAs) of the electron donors and acceptors, respectively, by DFT calculations. In electrochemistry, an endothermic SET has a negative Δ*E*_SET,CV_, while for exothermic SET processes the Δ*E*_SET,CV_ is positive. Thus, if Δ*E*_SET,CV_ is larger than −0.4 V, radicals can potentially be observed.

The determination of *E*_ox_ and *E*_red_ can be achieved experimentally by cyclic voltammetry (CV).^20,21^ CV also provides valuable information on the stability of the formed radicals. Radicals that are stable on the CV timescale (typically in the range of minutes) exhibit a reversible redox event with both the oxidation to the radical and reduction of the radical, or *vice versa*, being visible. Conversely, unstable radicals cannot be reduced or oxidised back to the closed-shell state, and therefore these species display only a single, irreversible redox event in CV. For reversible redox events the halfwave potential, *E*_1/2_, is taken as a measure of the redox potential, while for irreversible redox events the half peak potential, *E*_p/2_, is used.^20^

**Fig. 1:**
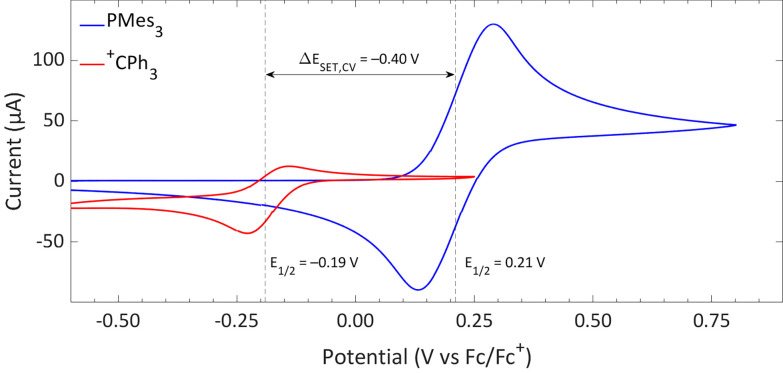
CVs of PMes_3_ and [CPh_3_][B(C_6_F_5_)_4_] in DCM with [*n*Bu_4_N][PF_6_] (0.5 M) as electrolyte at a scan rate of 100 mV s^−1^, showing a quasi-reversible and reversible redox event, respectively. Further experimental details are reported in the ESI.†

The CVs of the individual compounds of the first model system of this review, PMes_3_/[CPh_3_][B(C_6_F_5_)_4_], are displayed in Fig. 1. In the case of PMes_3_, a reversible redox event is observed, with an *E*_1/2_ of 0.21 V *vs.* Fc/Fc^+^.^22^ For the trityl cation (CPh_3_^+^), the redox event at −0.19 V *vs.* Fc/Fc^+^ is quasi-reversible, which is most likely due to the formation of the Gomberg dimer (CPh_3_)_2_ upon reduction, limiting the number of radicals that can be reoxidised.^23,24^ Combining the two redox potentials yields a slightly endothermic Δ*E*_SET,CV_ of −0.40 V, which predict a thermal SET to be feasible, as Klare, Müller and colleagues indeed observed, and as we will further highlight in Section 3.

**Fig. 2:**
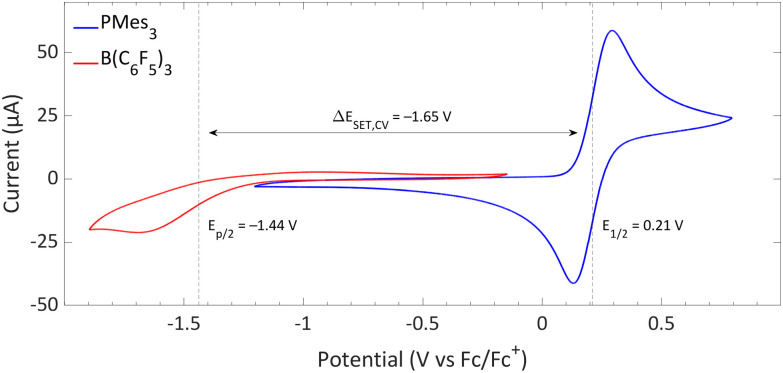
CVs of B(C_6_F_5_)_3_ and PMes_3_ in DCM at a scan rate of 100 mV s^−1^, showing an irreversible and reversible redox event, respectively. For B(C_6_F_5_)_3_, [*n*Bu_4_N][B(C_6_F_5_)_4_] (0.05 M) was used as electrolyte, while for PMes_3_ [*n*Bu_4_N][PF_6_] (0.5 M) was used. Experimental details are reported in the ESI.†

Switching the electron acceptor from CPh_3_^+^ to the weaker electron acceptor B(C_6_F_5_)_3_ changes the Δ*E*_SET,CV_ accordingly, as shown in Fig. 2. The CV of B(C_6_F_5_)_3_ in DCM, as reported by Wildgoose, showed an irreversible reduction due to the decomposition of the formed B(C_6_F_5_)_3_˙^−^ radical anion *via* solvolysis reactions.^25^ Compared to [CPh_3_][B(C_6_F_5_)_4_], B(C_6_F_5_)_3_ has a 1.37 V lower reduction potential (–1.44 V *vs.* Fc/Fc^+^ for B(C_6_F_5_)_3_), resulting in an endothermic Δ*E*_SET,CV_ of −1.65 V. This significant potential difference indicates that a thermal SET is no longer feasible. On the other hand, in case of a photoinduced SET process, the absorption of visible light (*λ* = 400–800 nm, Δ*E* = 71.4 to 35.7 kcal mol^−1^) can induce a SET when the radical pair is approximately 1.5 to 3.1 eV higher in energy than the closed-shell state.^8^ For radical pairs that are between 0.4 and 1.5 eV higher in energy than the closed-shell state, likely infrared irradiation is required for the SET to occur. With the couple PMes_3_/B(C_6_F_5_)_3_, the found Δ*E*_SET,CV_ of −1.65 V falls within the range of visible light, predicting a photoinduced SET to be feasible.^13^ Indeed, the photoinduced nature of the SET from PMes_3_ to B(C_6_F_5_)_3_, resulting in the radical ion pair PMes_3_˙^+^/B(C_6_F_5_)_3_˙^−^, has been confirmed by us and will be discussed in Section 3.

While cyclic voltammetry can predict the feasibility of thermal and photoinduced SET events, its accuracy is highly sensitive to experimental conditions, such as changes in solvent, electrolyte, and electrodes between measurements. This sensitivity hampers the ability to accurately compare reported redox potentials between electron donors and acceptors. Furthermore, specific solvents, such as toluene or 2-methyltetrahydrofuran, are exceptionally suitable for studying radicals at low temperatures by EPR spectroscopy. However, apolar solvents like toluene are not suitable for CV due to their limited ability to dissolve the required amount of electrolyte (typically 0.1–0.5 M of [*n*Bu_4_N][PF_6_]) for adequate conductivity. Therefore, DCM or acetonitrile is often used for electrochemical studies, even though the obtained Δ*E*_SET,CV_ can be inaccurate due to more extensive stabilisation of the charged state in more polar solvents.

An alternative to determining redox properties by CV measurements is the *in silico* determination of the ionisation energies (IEs) and electron affinities (EAs) of the electron donors and acceptors, respectively, by DFT calculations. These values are obtained by comparing the computed relative energy of the closed-shell ground state with the singly oxidised/reduced radical state and, by the summation of the IE and EA, the Δ*E*_SET,calc_ is obtained. Corrections for solvent effects can be implemented using, for example, self-consistent reaction field (SCRF) methods (*e.g.*, the polarizable continuum model).^26^ This approach allows the estimation of redox properties in a wide variety of solvents, even those problematic for CV measurements.

**Fig. 3:**
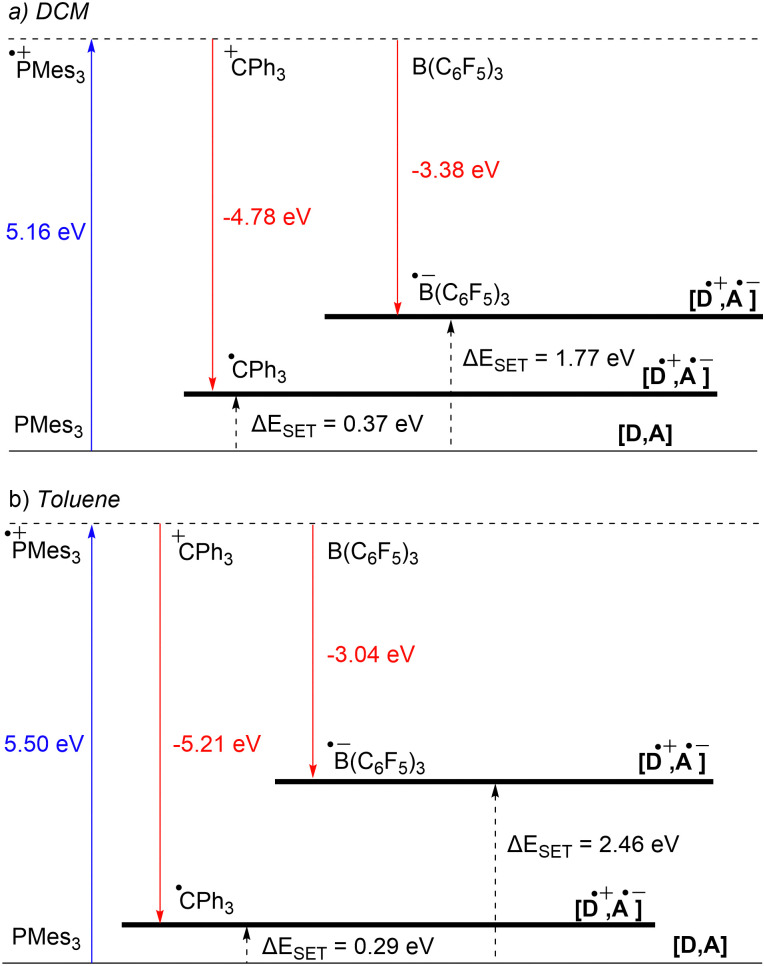
Energy diagram with calculated ionisation energy (IE) of PMes_3_ (blue) and electron affinities (EA) of CPh_3_^+^ and B(C_6_F_5_)_3_ (both red) for both (a) DCM and (b) toluene as the solvent. For both the photoinduced SET (PMes_3_, B(C_6_F_5_)_3_) and thermally induced SET (PMes_3_, CPh_3_^+^) the Δ*E*_SET,calc_ are indicated (black arrows).

Starting with the PMes_3_/CPh_3_^+^ system, calculations at the SCRF(DCM)/(U)*ω*B97X-D/6-311+G(d,p)//(U)*ω*B97X-D/6-31G(d) level of theory afford an IE of 5.16 eV for PMes_3_ and an EA of −4.78 eV for CPh_3_^+^.^13^ As visualised in Fig. 3a, this results in a Δ*E*_SET,calc_ of 0.37 eV in DCM, indicating, in agreement with the electrochemically found Δ*E*_SET,CV_ of −0.40 V, that a thermal SET is feasible. For PMes_3_/B(C_6_F_5_)_3_, calculations predict only a photoinduced SET to be feasible, with an EA of −3.38 eV for B(C_6_F_5_)_3_, resulting in a Δ*E*_SET,calc_ of 1.77 eV. This finding is also in agreement with CV measurements, where a Δ*E*_SET,CV_ of −1.65 V was found.

Due to the use of a solvent model in the calculations, the influence of the solvent on the Δ*E*_SET,calc_ can be readily computed. Since the SET process always involves charged species, solvent polarity significantly impacts the calculated IEs and EAs. For a neutral electron donor and acceptor, the charges of the corresponding RIP will be more stabilised in polar solvents, leading to a more favourable Δ*E*_SET_. This phenomenon is clearly evident for PMes_3_/B(C_6_F_5_)_3_, where the Δ*E*_SET,calc_ increases from 1.77 eV to 2.46 eV when switching from DCM to the less polar toluene (Fig. 3b). In the case of PMes_3_/CPh_3_^+^, a reverse trend is observed (0.37 eV in DCM *vs.* 0.29 eV in toluene), which is attributed to the fact that CPh_3_^+^ is charged and, therefore, relatively more stabilised in polar solvents than its trityl radical counterpart (CPh_3_˙) formed after SET. As the characterisation of the radical pairs is performed in toluene (see Section 3), the Δ*E*_SET_ based on the DFT calculations for these systems provides a better indicator than the ones based on CV data measured in DCM.

### 2.2. Characterisation of the EDA of a photoinduced SET system

**Fig. 4:**
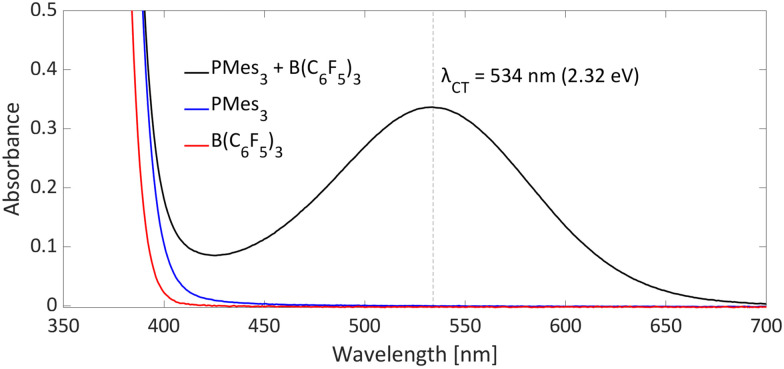
UV-vis spectrum of toluene solutions of PMes_3_ (45 mM; blue), B(C_6_F_5_)_3_ (30 mM; red) and the combination of PMes_3_ (45 mM) and B(C_6_F_5_)_3_ (30 mM; in black).

Typical for photoinduced SET systems is the emergence of a new band in the UV-vis spectrum upon mixing the electron donor and electron acceptor in solution. This so-called charge-transfer (CT) band is often found at longer wavelengths than the absorption of the individual components. We reported that, in the case of PMes_3_/B(C_6_F_5_)_3_, such a CT-band exist, giving rise to the violet colour of the solution and originates from the FLP encounter complex, which is the electron donor–acceptor (EDA) complex [PMes_3_, B(C_6_F_5_)_3_].^13^ For a toluene solution of PMes_3_/B(C_6_F_5_)_3_, the UV-vis spectrum, displayed in Fig. 4, clearly shows the presence of a CT-band at 534 nm (2.32 eV), as the individual components do not absorb in this region. Upon irradiation of this new band, SET occurs from the electron donor to the acceptor, affording the transient RIP PMes_3_˙^+^/B(C_6_F_5_)_3_˙^−^. Ideally, the CT-band is irradiated specifically where the individual components do not absorb, to exclude effects from donor and acceptor excited states. The energy corresponding to the *λ*_max_ at 534 nm (2.32 eV) correlates well with the in toluene calculated Δ*E*_SET,calc_ of 2.46 eV (504 nm), indicating a small electronic coupling term (*ω*) and, therefore only a weak interaction between the donor and acceptor, indicative of low concentrations of the EDA complex [PMes_3_, B(C_6_F_5_)_3_] in solution. Marques and Ando recorded the UV-vis spectrum of PMes_3_/B(C_6_F_5_)_3_ in DCM and found the CT-band at 526 nm (2.36 eV), a small blue shift of 8 nm compared to toluene as the solvent.^27^ On the other hand, the obtained Δ*E*_SET_ values in DCM (Δ*E*_SET,CV_ = −1.65 V and Δ*E*_SET,calc_ = 1.77 eV) predicted a significantly larger blue shift compared to the Δ*E*_SET,calc_ in toluene, which was expected due to the higher amount of stabilisation of the ions by the solvent in DCM. This anticipated discrepancy between Δ*E* and *λ*_CT_ is likely attributable to variations in interaction between the electron donor and acceptor across different solvents. These variations lead to differing degrees of orbital mixing and their energies, resulting in distinct values of *ω* in Mulliken theory.^14,15^ For this reason, the estimation of Δ*E*_SET_ can only be used as a guiding tool to discriminate between thermal and photoinduced processes, and for photoinduced SET processes, the exact excitation wavelength should be determined by measuring the UV-vis spectrum and subsequent determination of the *λ*_max_ of the charge-transfer band.

Besides experimentally observing the CT-band with UV-vis spectroscopy, time-dependent DFT calculations (TD-DFT) can be used to estimate the excitation wavelength for the SET to occur. We calculated the wavelength of the CT-band of the EDA complex [PMes_3_, B(C_6_F_5_)_3_] to be *λ*_CT,calc_ = 439 nm (2.82 eV) in the gas phase (*ω*B97X-D/6-311++G(d,p)//*ω*B97X-D/6-311G(d,p) level of theory). As solvent effects were not included, the calculated value is an overestimation of the experimentally observed value by UV-vis (*λ*_CT_ = 534 nm, 2.32 eV), due to the stabilisation of the RIP by the solvent. Satisfactorily, when Marques and Ando applied a solvent correction for DCM, they found an excitation energy of 2.19 eV (565 nm, at the *ω*B97X-D/6-31G(d,p) level of theory), which is closer to the experimentally observed value in DCM (*λ*_max_ = 526 nm, 2.36 eV).^27^ In addition to the excitation wavelength of the EDA complex, TD-DFT provides insight into the frontier molecular orbitals that are involved. For PMes_3_/B(C_6_F_5_)_3_, the TD-DFT calculations indicate that the SET occurs from the formal lone pair of PMes_3_ (HOMO) to the formal vacant orbital of the borane (LUMO), confirming direct electron transfer from the electron donor to the electron acceptor.^13,27^

Absorption spectroscopy can yield information not only about the required wavelength for the SET to occur but also about the concentration of the EDA complex in solution. Since the EDA complex is in equilibrium with the separate electron donor (Lewis base) and electron acceptor species (Lewis acid), it has an association constant (*K*_a_), as shown in Scheme 1. To determine *K*_a_, one can use the *λ*_max_ absorption of the CT-band and the Benesi–Hildebrand equation:^28^
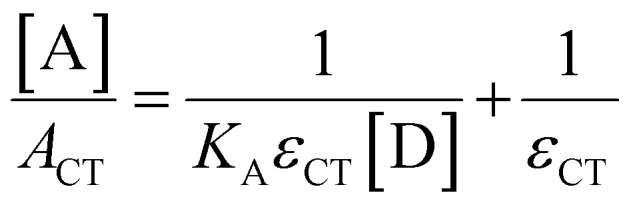


By using an excess of electron acceptor compared to the electron donor, and varying the concentration of the acceptor [A] (or *vice versa*), both the absorption coefficient of the EDA *ε*_CT_ and the *K*_a_ can be calculated. Although this method is straightforward in its application, it can be unreliable due to oversimplifications, for example, in approximating the actual concentration of the free donor or acceptor to the initial concentration before complexation.^29^ Instead, the use of modern non-linear fitting methods developed in supramolecular chemistry is preferred, as demonstrated by Jupp and colleagues for the combination PMes_3_ and B(C_6_F_5_)_3_ in toluene.^30^ They obtained a *K*_a_ of 2.52 ± 0.43 M^−1^ using a constant B(C_6_F_5_)_3_ concentration (5 mM), but varying PMes_3_ concentration (5–300 mM). This association constant corresponds to a Δ*G* of −0.55 kcal mol^−1^, which suggests that 1.2% of the individual Lewis base and Lewis acid are present in the EDA complex [PMes_3_, B(C_6_F_5_)_3_] in solutions containing 5 mM of each component. Interestingly, by solely increasing the ratio PMes_3_:B(C_6_F_5_)_3_ ratio from 1 : 1 to 10 : 1 (while maintaining the concentration of B(C_6_F_5_)_3_ at 5 mM), 11.1% of the borane is captured in the EDA complex. These results demonstrate that by varying the donor : acceptor ratio, a greater proportion of the individual components can be incorporated in the EDA complex, thereby influencing the reaction kinetics for the activation of small molecules.

### 2.3 Origin of colour: charge transfer band *vs.* radicals

Since radicals are typically highly coloured (*e.g.*, bulky PAr_3_˙^+^ radicals have an absorption in the region of 500–600 nm, depending on the solvent and counterion),^31,32^ it is crucial to accurately determine the origin of the colour of the reaction mixture, and thus to distinguish between colour stemming from the presence of radicals or the charge transfer band (EDA complex). For example, in early work, Stephan *et al.* attributed the purple colour of toluene solutions of PMes_3_/Al(C_6_F_5_)_3_ and PMes_3_/B(C_6_F_5_)_3_ to the presence of radicals.^31^ Namely, for PMes_3_/Al(C_6_F_5_)_3_, the solution is deep purple, while the authors observed a strong signal for the PMes_3_˙^+^ radical cation by EPR. Conversely, a solution of PMes_3_ and the weaker Lewis acid B(C_6_F_5_)_3_ is only pale purple, and Stephan *et al.* observed only weak EPR signals for PMes_3_˙^+^. We reported that the encounter complex is also coloured due to its CT-band in the visible spectrum. Therefore, depending on the experimental conditions and specific donor–acceptor system, the observed colour of the reaction mixture can stem from the EDA complex formed in the dark or the colour of persistent radicals formed upon photoinduced SET. In this example, the PMes_3_/LA mixtures of Stephan *et al.* were prepared in broad daylight, facilitating the formation of the PMes_3_˙^+^ radical cation after a photoinduced SET, whose concentration increased over time due to the decomposition of the fleeting Al(C_6_F_5_)_3_˙^−^ or B(C_6_F_5_)_3_˙^−^ radical anion.^13^ This shows that careful interpretation of the colour of solutions containing an electron donor–acceptor pair is required.

## 3. Characterisation of the radical (ion) pair

After predicting whether a SET occurs thermally or is photoinduced, the next step is to confirm this by characterising the formed radical pairs under conditions specific to each type of SET. Accordingly, we will now focus on the differences in characterisation methods required for both thermally and photochemically formed radical pairs. First, electron paramagnetic resonance (EPR) spectroscopy is discussed, as it is applicable to both types of SET. Subsequently, we will focus on techniques specific to each type of SET, beginning with UV-vis spectroscopy for the characterisation of thermally induced radical pairs. This will be followed by the use of transient absorption and resonance Raman spectroscopy, which are valuable methods for elucidating photoinduced SET events.

Note: In this section, we will focus solely on the simultaneous characterisation of both radicals of the pair. However, when one of the two radicals exhibits a limited lifetime, Le Chatelier's principle dictates that the other, more persistent radical will accumulate.^33^ Consequently, this leads to the characterisation of only a single radical, instead of the entire radical pair.

### 3.1. Electron paramagnetic resonance (EPR) spectroscopy

#### 3.1.1. Thermal single electron transfer

**Fig. 5:**
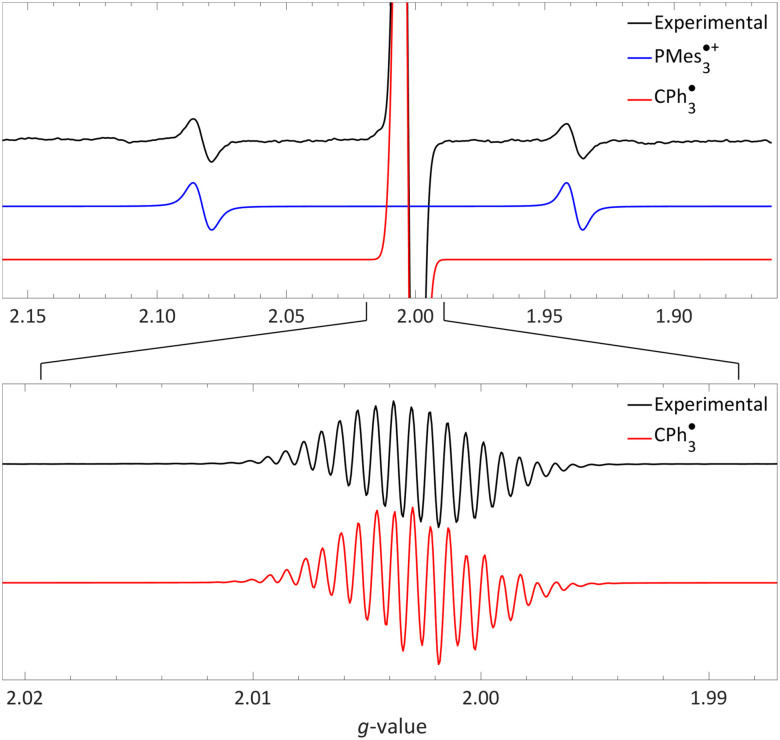
Room temperature EPR of PMes_3_/[CPh_3_][B(C_6_F_5_)_4_] in toluene, yielding the thermal radical pair consisting of PMes_3_˙^+^ (*g*_iso_ = 2.0022, ^31P^*a*_iso_ = 41.0 mT) and CPh_3_˙ (*g*_iso_ = 2.0028, ^1H,o^*a*_iso_ = 0.26 mT, ^1H,m^*a*_iso_ = 0.11 mT, ^1H,p^*a*_iso_ = 0.28 mT). The bottom spectrum is a zoom in, under different measurement conditions, of the top spectrum. Further experimental and simulation details are reported in the ESI.†

For a thermal SET, the radical pair is directly formed upon mixing the electron donor and acceptor and can be observed by EPR spectroscopy at room temperature if both radicals are persistent under these conditions. For the system PMes_3_/[CPh_3_][B(C_6_F_5_)_4_] in toluene, the room temperature EPR spectrum (black trace in Fig. 5) indeed shows both radicals to be present, as reported by Klare, Müller and colleagues.^12,13^ This observation aligns with the slightly exothermic Δ*E*_SET,CV_ = −0.40 V and Δ*E*_SET,calc_ = 0.29 eV, alongside the known thermal stability of PMes_3_˙^+^ and the trityl radical.^34,35^ In this context, the formation of the Gomberg dimer (CPh_3_)_2_ shifts the ED/EA−radical pair equilibrium further towards the radical side by 4.7 kcal mol^−1^ (0.20 eV).^13,23,24^ The PMes_3_˙^+^ radical cation is characterised by its large isotropic phosphorus hyperfine coupling (^31P^*a*_iso_) of 670 MHz (23.9 mT, *g*_iso_ = 2.0054; blue trace).^31,32^ The signal at *g*_iso_ = 2.0028 is assigned to CPh_3_˙, which, upon closer examination (lower graph, red trace), reveals a rich hyperfine structure stemming from coupling to the phenyl hydrogen atoms.^36^ Simulations of the experimental spectra indicate that the ratio between PMes_3_˙^+^ and CPh_3_˙ is 1 : 6.8. This discrepancy could be attributed to the formation of a biphasic system, as observed by Klare and Müller and colleagues in benzene. The lower, ionic phase contained solely the phosphonium radical cation, while the top, non-polar layer showed the presence of both the phosphonium radical cation and trityl radical, as the authors observed with EPR spectroscopy.^12^

The visibility of both the phosphine radical cation and trityl radical at room temperature *via* EPR spectroscopy, without explicit irradiation during measurement, supports a thermal SET. However, inherent light from the surroundings can also induce radical formation *via* a photoinduced SET, as for example is the case in the seminal contribution of Wang *et al.*^32^ Therefore, as definitive proof that for PMes_3_/[CPh_3_][B(C_6_F_5_)_4_] SET occurs thermally, we measured the EPR spectrum both with and without ambient light and found that in both instances the same EPR spectrum was obtained.^31^ Thus, it is conclusively established that the SET for the PMes_3_/CPh_3_^+^ system occurs thermally.

#### 3.1.2. Photoinduced single electron transfer

For a photoinduced SET, as reported for PMes_3_/B(C_6_F_5_)_3_ (Δ*E*_SET,CV_ = −1.65 V, Δ*E*_SET,calc_ = 2.46 eV), observing the high energy, transient RIP PMes_3_˙^+^/B(C_6_F_5_)_3_˙^−^ by EPR requires irradiation of the charge transfer band of the EDA complex [PMes_3_, B(C_6_F_5_)_3_]. The ground state of the EDA complex is a singlet, hence the obtained radical pair will also be in the singlet state, as only this transition is permitted according to Mulliken.^15^ Additionally, the corresponding back-electron transfer (BET) from the singlet RIP back to the singlet EDA ground state is allowed; therefore, the likelihood of facile BET should always be considered. Owing to facile BET, the radical pair's lifetime can be so brief that it impedes observation at room temperature. For example, in the case of PMes_3_/B(C_6_F_5_)_3_, the half-life of the RIP at room temperature is only 237 ps, as determined by transient absorption spectroscopy (see Section 3.3.1).^13^

**Fig. 6:**
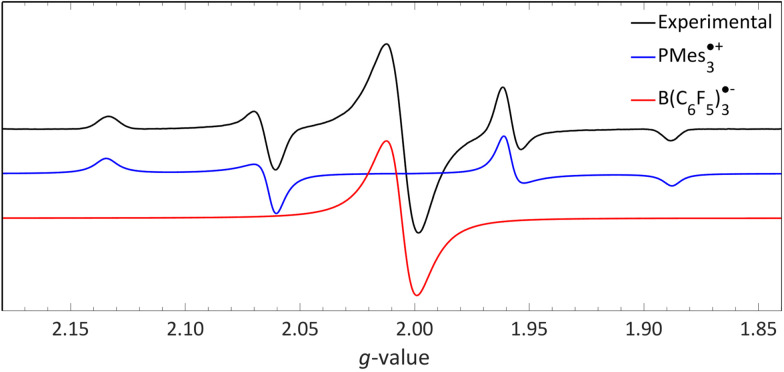
A 30 K EPR spectrum of PMes_3_ and B(C_6_F_5_)_3_ using 390–500 nm irradiation yielding photoinduced SET to afford the RIP consisting of PMes_3_˙^+^ (*g*_⊥_ = 2.0050, *g*_‖_ = 2.0022, ^31P^*a*_⊥_ = 17.0 mT, ^31P^*a*_‖_ = 41.0 mT) and B(C_6_F_5_)_3_˙^−^ (*g*_iso_ = 2.0057). Further experimental and simulation details are reported in the ESI.†

We recorded EPR spectra for the combination of PMes_3_ and B(C_6_F_5_)_3_ in frozen toluene glass at 30 K.^13^ Before irradiation, no signals were observed, confirming that the violet colour of PMes_3_/B(C_6_F_5_)_3_ solutions in the dark originates from the EDA complex [PMes_3_, B(C_6_F_5_)_3_]. However, upon direct irradiation of the EPR tube in the spectrometer at 30 K with 390–500 nm light, a multisignal spectrum was obtained (Fig. 6, black trace). This photoinduced event generated the RIP PMes_3_˙^+^/B(C_6_F_5_)_3_˙^−^, as evidenced by the spectrum featuring both radicals. The signal for PMes_3_˙^+^ transformed from an isotropic doublet recorded in solution (blue trace in Fig. 5) into a more complex, four line axial signal at 30 K (blue trace in Fig. 6), due to anisotropy in the solid toluene matrix from reduced tumbling of the radical. The observed large phosphorus hyperfine couplings of ^31P^*a*_⊥_ = 477 MHz (17.0 mT) and ^31P^*a*_‖_ = 1149 MHz (41.0 mT) align with reported low-temperature EPR spectra of PMes_3_˙^+^.^37^ The remaining broad, featureless singlet at g = 2.0057 was assigned to the B(C_6_F_5_)_3_˙^−^ radical anion (red trace). The width of the signal matches the spectra of B(C_6_F_5_)_3_˙^−^ in THF at −50 °C, as reported by Norton and colleagues,^38^ although in their study, hyperfine couplings with boron and fluorine were resolved. Simulations confirm that both radicals were formed in equal amounts, verifying the stoichiometry of this SET process. This demonstrates that low-temperature EPR is a powerful tool for detecting short-lived radicals, such as B(C_6_F_5_)_3_˙^−^.^38^

### 3.2. UV-vis spectroscopy for thermal radical pair characterisation

**Fig. 7:**
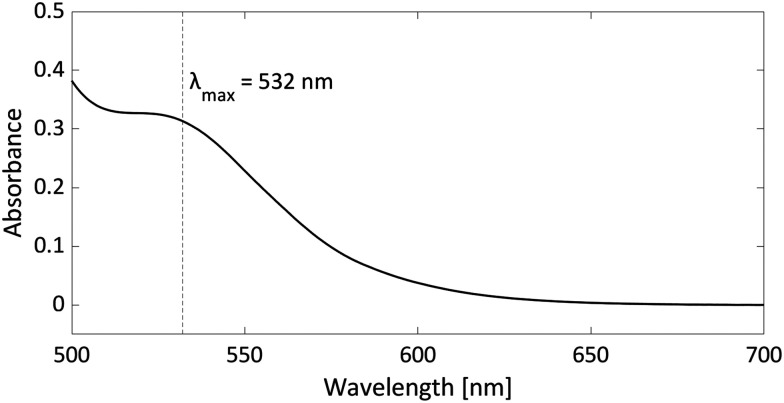
UV-vis spectrum of the radical pair consisting of pTipp_3_˙^+^ and CPh_3_˙ in chlorobenzene with a concentration for both of 2.25 mM.

As radicals are typically highly coloured species with strong absorptions in the visible spectrum, UV-vis spectroscopy can provide additional evidence for the presence of radicals, particularly in the case of thermally accessible radical pairs. For the model system PMes_3_/[CPh_3_][B(C_6_F_4_)_4_], no UV-vis data are reported; however, for the related pTipp_3_/[CPh_3_][B(C_6_F_4_)_4_] (Tipp = 2,4,6-iPr_3_C_6_H_2_) in chlorobenzene, the UV-vis spectrum shows a broad absorption band with *λ*_max_ = 532 nm (Fig. 7).^12^ The trityl radical CPh_3_˙ is characterised by an absorption at *λ*_max_ = 510 nm,^36^ while for the phosphoniumyl radical pTipp_3_˙^+^*λ*_max_ = 539 nm is a typical value.^12^ Due to the close proximity of the absorption maxima of both radicals, the absorption band cannot be assigned to specific radicals. However, it shows that UV-vis spectroscopy can be used alongside EPR spectroscopy to indicate the presence of coloured radical species.

### 3.3. Characterisation of a photoinduced radical pair

#### 3.3.1. Transient absorption spectroscopy

In case of a photoinduced SET, no radicals are present without irradiation; therefore, UV-vis spectroscopy cannot be used to observe the radical pair. Instead, transient absorption spectroscopy proves to be useful, where the first excitation pulse, known as the pump pulse, irradiates the CT-band, yielding the radical pair.^39^ The radical pair can then be observed during the second pulse, or probe pulse, where a full UV-vis spectrum is obtained using a single pulse. By varying the delay of the second pulse after the first, information on the lifetime of the radical pair can be obtained.

**Fig. 8:**
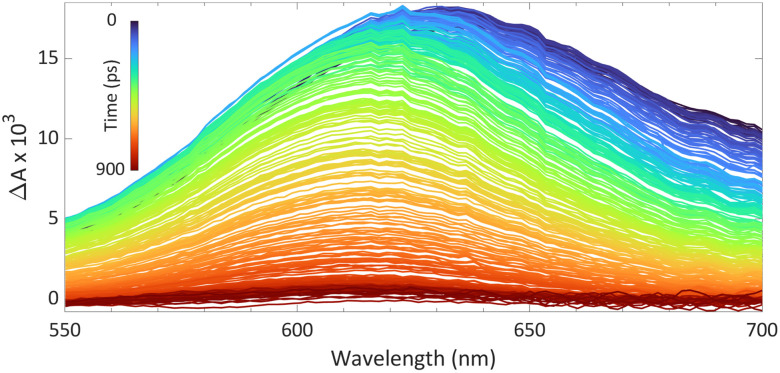
Room temperature transient absorption spectrum of PMes_3_ and B(C_6_F_5_)_3_ in toluene using a 530 nm excitation pulse (<200 fs).

We reported using this technique to study PMes_3_/B(C_6_F_5_)_3_ in toluene at room temperature (Fig. 8).^13^ Using a 530 nm pump pulse (<200 fs) to irradiate the CT-band, the FLP is excited to the RIP PMes_3_˙^+^/B(C_6_F_5_)_3_˙^−^. The subsequent probing pulse revealed the formation of a new broad absorption band around 620 nm, assigned to the absorption of both PMes_3_˙^+^ (573–600 nm)^31,40^ and B(C_6_F_5_)_3_˙^−^ (+/− 600 nm).^38^ Time-resolved measurements indicated that the half-life of the RIP absorption was 237 ps, due to rapid back electron transfer (BET) to the closed-shell ground state. This rapid BET also hinders follow-up chemistry of this radical ion pair, as its short-lived nature does not allow for any intermolecular reactions with substrates that require lifetimes on the order of milliseconds.^41^ Instead, decomposition of one of the radicals can provide a competitive pathway to BET, leading to the accumulation of the more persistent radical. Indeed, as B(C_6_F_5_)_3_˙^−^ is known to decompose quickly,^38^ an increasing concentration of PMes_3_˙^+^ over time is typically observed, supported by an intensifying EPR signal and colouring of the solution. The isolation of solely the triarylamine radical cation by Wang *et al.* (Scheme 2) also exemplifies this concept.^32^

#### 3.3.2. Resonance Raman spectroscopy

Ando and colleagues utilised resonance Raman spectroscopy to confirm the involvement of both PMes_3_ and B(C_6_F_5_)_3_ in the photoinduced SET process upon irradiation of the CT-band.^27^ They measured the Raman spectrum of a DCM solution of PMes_3_ and B(C_6_F_5_)_3_ with two different excitation wavelengths (*λ*_exc_ = 497 and 1064 nm). While the Raman spectrum measured at *λ*_exc_ = 1064 nm shows no significant changes compared to isolated PMes_3_ and B(C_6_F_5_)_3_, indicating little interaction between the Lewis base and acid, the resonance Raman spectrum using *λ*_exc_ = 497 nm to irradiate the CT-band exhibits an enhancement of several vibrational bands; notably, two of the bands are at 857 and 1058 cm^−1^. With the help of computed Raman spectra, the authors assigned these bands to both PMes_3_ (*e.g.*, 1058 cm^−1^ to *ν*(C–P) and *ν*(C–C) stretching's and *δ*(C–C–C) bending) and B(C_6_F_5_)_3_ (*e.g.* 857 cm^−1^ to *ν*(B–C) and *ν*(C–F) stretching's). As the enhanced signals could be attributed to both the Lewis base and the Lewis acid, this unequivocally demonstrates that both species participate in the charge transfer process that occurs upon irradiation of the CT-band.

## 4. Beyond FLPs: application of single electron transfer to main-group (photo)redox chemistry

Electron donor–acceptor systems often display behaviours that complicate spectroscopic characterization, such as the facile decomposition of radicals formed following SET. Therefore, it is often necessary to conduct additional experiments to allow full characterisation of the radical pair. This section is dedicated to examining three particular case studies of well researched systems, along with the experiments conducted by the authors to characterize the radicals. Through these examples, attention is given to both thermal and photoinduced single electron transfer (SET), as well as the employment of Lewis acid activation to facilitate electron acceptance. The first system consists of lithium bis(trimethylsilyl)amide (LiHMDS)/[TEMPO][BF_4_] that, after thermal SET, affords the radical pair HMDS˙/TEMPO˙, which can be used for the functionalisation of C–H bonds in aliphatic substrates.^16,42^ Secondly, the (*p*BrPh)_3_N/tetracyanoquinodimethane (TCNQ) pair will be discussed that, after thermal SET from the N-based electron donor to TCNQ, affords a radical ion pair.^17^ In this system, the coordination of B(C_6_F_5_)_3_ to TCNQ will be emphasized as an effective method for enhancing SET, by modifying the electron-accepting properties of TCNQ. The third case study features a sulfonium cation as the electron acceptor in a photoinduced SET process.^18^ This SET process results in the formation of a CF_3_ radical through the homolytic cleavage of the reduced sulfonium salt, which is then utilized for the trifluoromethylation of a range of substrates.

### 4.1. C–H functionalisation using the thermally induced radical Pair HMDS˙/TEMPO˙

**Scheme 4:**
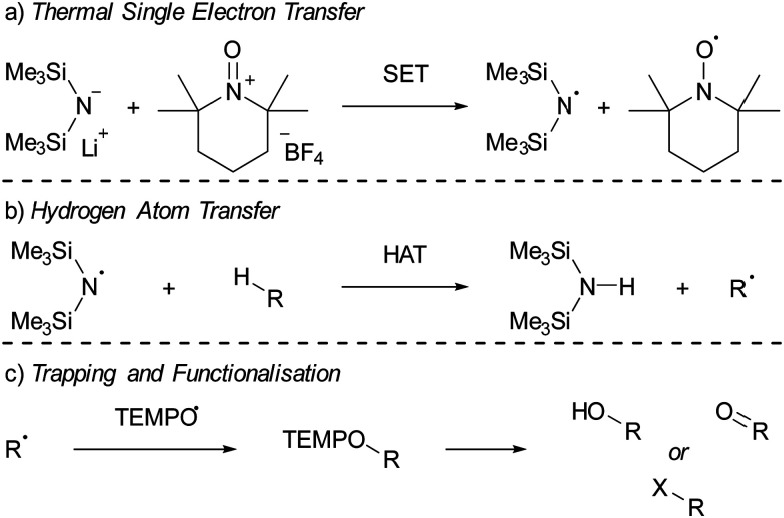
The C–H functionalisation using LiHMDS and [TEMPO][BF_4_] as reported by Lin *et al.* (a) thermal single electron transfer to yield the HMDS˙/TEMPO˙ radical pair. (b) Subsequent HAT from a substrate by HMDS˙ and (c) trapping of the resulting radical by TEMPO˙. R = a carbon centred group; X = F, Cl, D, or various other nucleophiles.

Lin and colleagues pioneered an innovative C–H activation reaction through the *in situ* formation of the radical pair HMDS˙/TEMPO˙ (Scheme 4).^16,42^ The suggested mechanism initiates with a thermal SET from the electron donor HMDS^−^ to the TEMPO^+^ acceptor, affording the reactive HMDS˙/TEMPO˙ couple (Scheme 4a). Subsequently, a hydrogen atom transfer (HAT) from a C–H bond of different aliphatic substrates to the N-centred radical HMDS˙ can take place, leading to the formation of a robust N–H bond (BDE = 109 kcal mol^−1^, Scheme 4b). Then, the TEMPO˙ radical captures the emerging carbon-centred radical, forming a weak C–O adduct (BDE = 49 kcal mol^−1^, Scheme 4c).^43^ This relatively weak C–O bond in the TEMPO adduct facilitates additional functionalisation into a range of functional groups, including alcohols and ketones. Switching the Lewis base from LiHMDS to the sterically more crowded lithium hexaphenyldisilazide (LiHPDS) altered the regioselectivity towards less crowded C–H bonds. Conversely, employing the less bulky potassium *tert*-butoxide (KO^*t*^Bu) as the electron donor shifted the regioselectivity in favour of more sterically hindered C–H bonds.

#### 4.1.1. Redox potentials

**Fig. 9:**
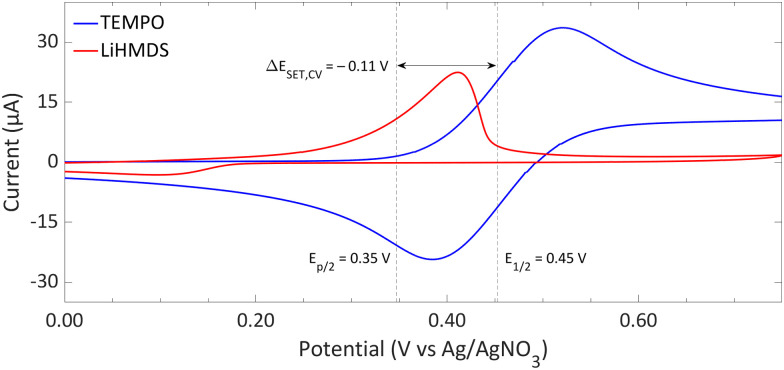
CVs of TEMPO and LiHMDS in *o*-difluorobenzene with [^*n*^Bu_4_N][PF_6_] (0.2 M) as supporting electrolyte at a scan rate of 100 mV s^−1^, showing an irreversible and reversible redox event, respectively. For clarity, the CVs are shown partly. The full spectra and experimental details are reported in the ESI.†*E*_1/2_ (Fc/Fc^+^) = +0.22 V *vs.* Ag/AgNO_3_.

We will first examine the redox potentials derived from CV measurements in *ortho*-difluorobenzene to ascertain the characteristics of the SET. Fig. 9 illustrates that the electron donor LiHMDS undergoes an irreversible oxidation, characterised by a half-peak potential of 0.35 V *vs.* Ag/AgNO_3_ (0.13 V *vs.* Fc/Fc^+^), which indicates the significant reactivity of the radical produced upon oxidation.^16^ On the other hand, TEMPO exhibits a reversible redox feature, signifying that both the radical and the cation remain stable under the tested conditions. Hence, employing TEMPO or TEMPO^+^ leads to an identical CV spectrum, since neither the TEMPO˙ nor TEMPO^+^ species undergoes decomposition. With the half-wave potential of 0.45 V *vs.* Ag/AgNO_3_ (0.23 V *vs.* Fc/Fc^+^) for TEMPO˙, the resulting Δ*E*_SET,CV_ of −0.11 V suggests that a thermal SET is feasible.

#### 4.1.2. Characterisation of the HMDS˙/TEMPO˙ radical pair

**Fig. 10:**
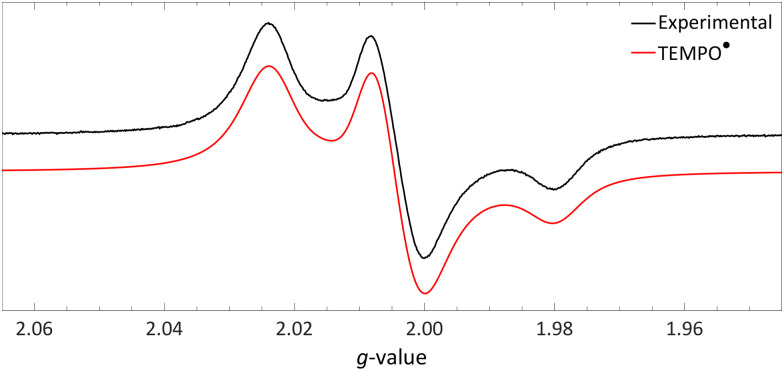
Obtained EPR spectrum of a flash frozen solution of LiHMDS and [TEMPO][BF_4_] to yield TEMPO˙ (*g*_⊥_ = 2.0067, *g*_‖_ = 2.0014, ^14N^*a*_⊥_ = 0.77 mT and ^14N^*a*_‖_ = 3.7 mT) in PhCF_3_. Further experimental and simulation details are reported in the ESI.†

To verify the formation of the HMDS˙/TEMPO˙ radical pair, Lin *et al.* conducted *in situ* EPR spectroscopy on a flash frozen trifluorotoluene solution containing [TEMPO][BF_4_] and LiHMDS (Fig. 10). The spectra reveals the generation of TEMPO˙ (*g*_⊥_ = 2.0067, *g*_‖_ = 2.0014, ^14N^*a*_⊥_ = 21.5 MHz (0.77 mT) and ^14N^*a*_‖_ = 104 MHz (3.7 mT)),^44^ thereby confirming the reduction of TEMPO^+^. Conversely, HMDS˙ was not detected, which the authors attribute to its rapid decomposition. As a result, complete characterisation of the radical pair *via* EPR proved to be unachievable, therefore the authors undertook additional experiments to demonstrate the formation of HMDS˙.

**Scheme 5:**
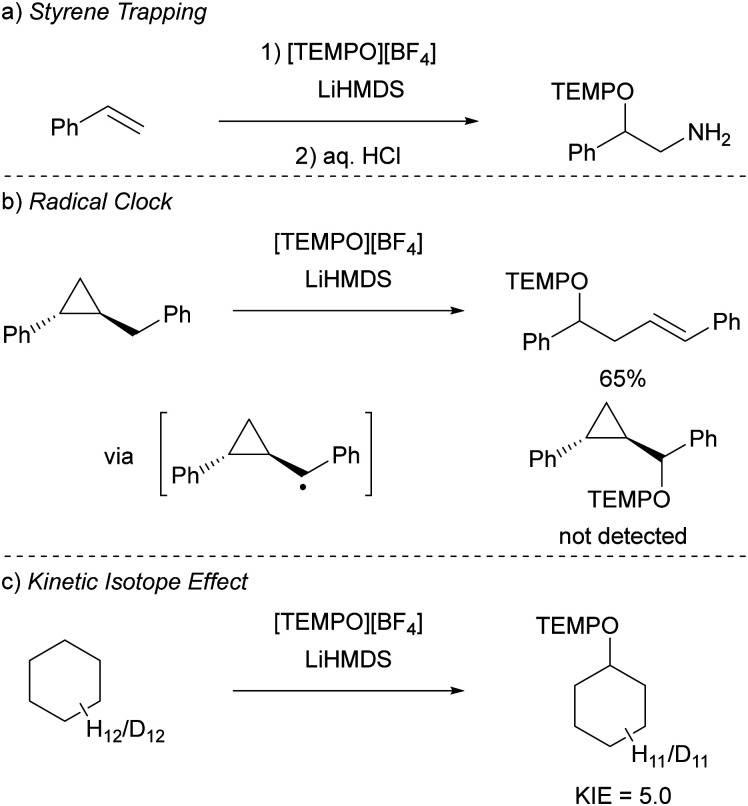
Mechanistic studies for the C–H functionalisation using the radical pair HMDS˙/TEMPO˙ as reported by Lin *et al.* (a) Trapping experiment using styrene showing the incorporation of (fragments of) the radicals. (b) Radical clock experiment with a cyclopropane substrate. (c) Intermolecular competition KIE experiment using cyclohexane.

Initially, to demonstrate the participation of both HMDS˙ and TEMPO˙ in the reaction, a trapping experiment was conducted using styrene as the trapping agent, resulting in the isolation of a difunctionalised TEMPO-amine product in 33% yield (Scheme 5a). The presence of components from both radicals (amine from HMDS˙ and TEMPO˙) in the product indicates their direct involvement in the reaction, although it does not alone verify the radical nature of this process. This was further investigated using a cyclopropane substrate in a radical clock experiment (Scheme 5b), where the only product obtained was the ring-opened product, while the corresponding non-ring-opened product was not observed. The rapid ring-opening (*k* = 3.6 × 10^8^ s^−1^ at 40 °C) following the generation of the benzylic radical confirms the radical mechanism of the C–H activation.^45^ Additionally, a primary kinetic isotope effect (KIE) of 5.0 for cyclohexane was observed, pinpointing C–H activation as the rate-determining step (Scheme 5c). DFT calculations supporting the proposed mechanism suggested a similar KIE, providing further evidence for the hypothesized HAT mechanism. The achieved regioselectivity by substituting LiHMDS with LiHPDS or KO^*t*^Bu highlights the role of HDMS^−^ in the crucial HAT step. Together with the radical nature confirmed by the aforementioned experiments, it can be concluded that HMDS˙ is indeed formed during the reaction, thus establishing the *in situ* formation of the HMDS˙/TEMPO˙ radical pair.

### 4.2. Promoting SET by increasing electron affinity *via* coordination of Lewis acids

The ability to tune the redox properties of compounds enhances the selection of more appropriate electron donors and acceptors, thus aiding electron transfer processes, promoting radical formation, and leading to the discovery of new radical pairs. The modification of redox potentials of compounds through the introduction of electron-donating and withdrawing groups is a well recognized strategy. However, the redox potentials of electron acceptors can be further refined by coordination with Lewis acids, which deplete electron density from the oxidant. This concept is for example demonstrated by Gray, Despagnet-Ayoub and colleagues, who illustrated that the oxidation potential of ferrocyanide (*E*_ox_ = −1.16 V *vs.* Fc^+^/Fc^0^) could be elevated by as much as 2.1 V to 0.85 V *vs.* Fc^+^/Fc^0^ through the coordination with six equivalents of B(C_6_F_5_)_3_.^46^ This finding builds on earlier work showing that solvent coordination to ferrocyanide similarly modulates the redox potential.^47^ Furthermore, a computational study by Thompson and Heiden revealed how the redox potential of benzoquinone could be adjusted with eight different Lewis acids (boranes and silylium cations).^48^ Additionally, the authors showed that even the coordination of a single proton has been found to shift the first redox potential by 1.33 V towards more positive values. Beyond Lewis acids, hydrogen bonding also influences redox potential changes, as demonstrated by Jacobsen, Nocera, and colleagues, who observed an increase of up to 0.63 V in redox potential of tetrachloro-*ortho*-quinone using a bis(amidinium) salt.^49^

**Scheme 6:**
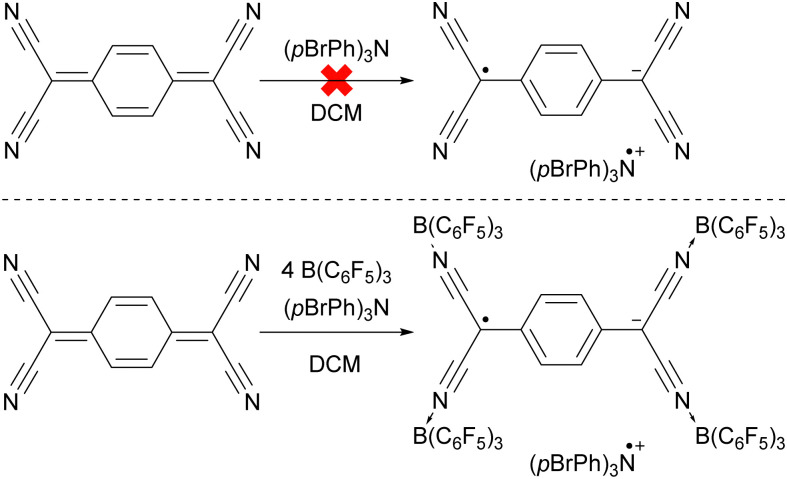
The thermal reduction of TCNQ using (*p*BrPh)_3_N as electron donor succeeds only using the coordination of B(C_6_F_5_)_3_ to TCNQ.

To emphasize the significance of Lewis acid coordination to facilitate SET processes, we will explore how tetracyanoquinodimethane (TCNQ) was modified through coordination with B(C_6_F_5_)_3_, as reported by Malischewski *et al.* (Scheme 6).^17^ In this instance, the coordination of four equivalents of the Lewis acid to the nitrogen atoms of TCNQ enhances its oxidizing ability, thereby enabling a thermal SET with (*p*BrPh)_3_N that results in the formation of a radical ion pair.

#### 4.2.1. Redox potentials

**Fig. 11:**
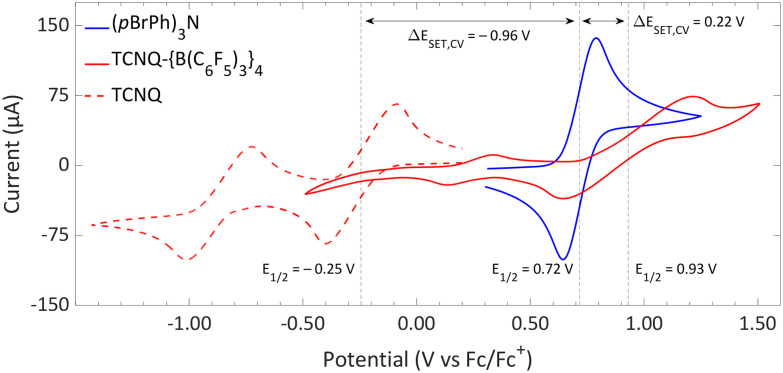
CVs of TCNQ-{B(C_6_F_5_)_3_}_4_, TCNQ and (*p*BrPh)_3_N in DCM, showing reversible redox events. For TCNQ and (*p*BrPh)_3_N [*n*Bu_4_N][PF_6_] (0.1 M) was added as electrolyte, while for TCNQ-{B(C_6_F_5_)_3_}_4_ no additional electrolyte was used. For clarity reasons part of the CV of TCNQ and (*p*BrPh)_3_N has been omitted. The full spectra and experimental details are reported in the ESI.†

CV measurements of TCNQ and its complex with four equivalents of B(C_6_F_5_)_3_, TCNQ-{B(C_6_F_5_)_3_}_4_, in DCM are shown in Fig. 11.^50^ Both exhibit two reversible reduction–oxidation events. The first, occurring at the highest potential, corresponds to the reduction of the neutral molecule to the radical anion, while the second event involves the formation of the dianion. The primary redox event, associated with the radical pair formation, has a half-wave potential of −0.25 V *vs.* Fc/Fc^+^ for TCNQ. This potential rises by approximately 1.2V to 0.93 V *vs.* Fc/Fc^+^ for TCNQ-{B(C_6_F_5_)_3_}_4_. The electron donor used, *p*(BrPh)_3_N, depicted in the same Fig. 11,^51^ has a half-wave potential of 0.72 V *vs.* Fc/Fc^+^, resulting in a Δ*E*_SET,CV_ of −0.96 V, when TCNQ serves as the electron acceptor. This value, being well beyond the −0.4 V threshold, suggest a thermal SET to be unlikely. However, a photoinduced SET could be plausible, with the energy difference equating to a wavelength of 1292 nm within the infrared spectrum. The coordination of four equivalents of B(C_6_F_5_)_3_ to form TCNQ-{B(C_6_F_5_)_3_}_4_ shifts the Δ*E*_SET,CV_ to a more favourable 0.22 V, implying a thermal SET to be feasible.

**Fig. 12:**
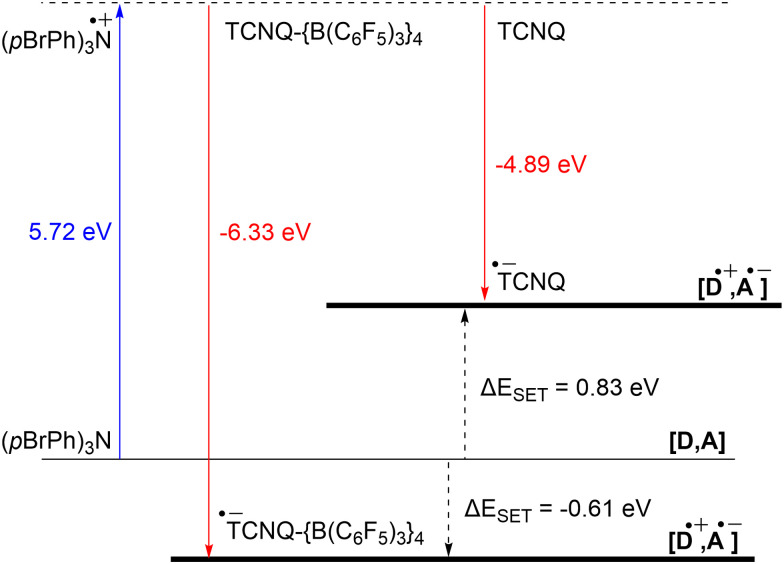
Energy diagram with calculated ionisation energy (IE) of (*p*BrPh)_3_N (blue) and electron affinities (EA) of TCNQ and TCNQ-{B(C_6_F_5_)_3_}_4_ (both red) for DCM. For both electron acceptors are the Δ*E*_SET,calc_ indicated (black arrows).

The significant impact of adding B(C_6_F_5_)_3_ is also evident in the ionization energy (IE) and electron affinity (EA) values calculated with DFT. For the (*p*BrPh)_3_N/TCNQ system, the calculated Δ*E*_SET,calc_ is 0.83 eV in DCM, indicating that a thermal SET is not favourable. However, a photoinduced SET, triggered by infrared light irradiation on the EDA complex [(*p*BrPh)_3_N, TCNQ] could be viable, aligning with the electrochemical findings. Similarly, the Δ*E*_SET,calc_ of −0.61 eV for (*p*BrPh)_3_N/TCNQ-{B(C_6_F_5_)_3_}_4_ suggests that, in this case, the SET process is thermally feasible in DCM (Fig. 12). This underscores the dramatic enhancement in EA upon coordination of four equivalents of B(C_6_F_5_)_3_ to TCNQ (from −4.89 eV to −6.33 eV), effectively lowering the energy of the radical ion pair below that of the closed-shell state.

#### 4.2.2. Characterisation of the RIP

**Fig. 13:**
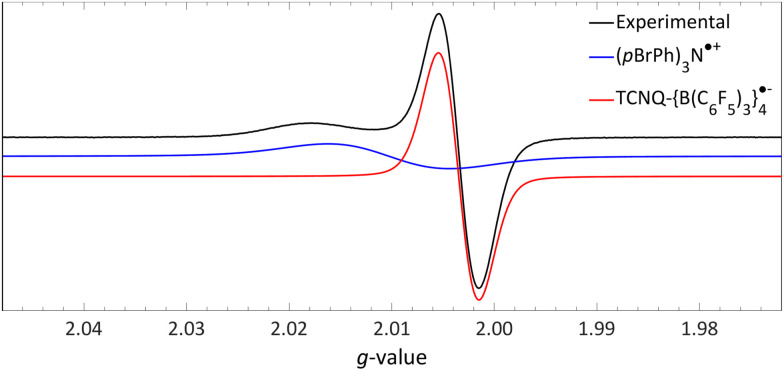
Room temperature EPR of the combination of TCNQ-{B(C_6_F_5_)_3_}_4_ (*g*_iso_ = 2.0035) and (*p*BrPh)_3_N (*g*_iso_ = 2.0103) in DCM. Further experimental and simulation details are reported in the ESI.†

The room temperature EPR spectrum of a DCM mixture containing both (*p*BrPh)_3_N and TCNQ-{B(C_6_F_5_)_3_}_4_ reveals the presence of two radicals, illustrated by the black line in Fig. 13. A distinct, relatively narrow signal at *g*_iso_ = 2.0035 (red line) is attributed to the radical anion of TCNQ-{B(C_6_F_5_)_3_}_4_, whereas a broader signal at *g*_iso_ = 2.0103 (blue line) corresponds to the (*p*BrPh)_3_N˙^+^ radical cation. The identification of TCNQ-{B(C_6_F_5_)_3_}_4_˙^−^ was validated through its independent production using ferrocene as reducing agent. Simulations further indicated that the ratio of the radical cation to radical anion closely matches 1 : 1, as expected for a RIP formed after a single electron transfer event, with no formation of the TCNQ dianion observed. To conclusively determine the necessity of B(C_6_F_5_)_3_ coordination to TCNQ for enabling thermal SET, it would be advisable to replicate the experiment using unmodified TCNQ, and also to explore the potential for photoinduced SET in the EDA complex [(*p*BrPh)_3_N, TCNQ].

### 4.3. Sulfonium salts utilized as electron acceptors in photoinduced SET processes

**Scheme 7:**
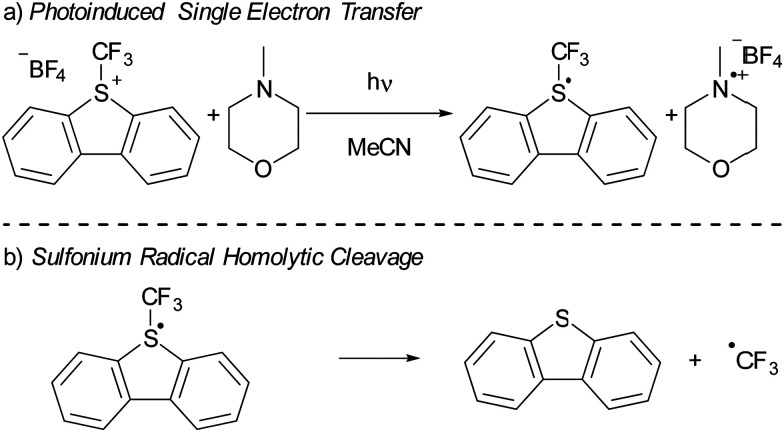
(a) The use of Umemoto's reagent (UR) as electron acceptor in a photoinduced SET with NMM as electron donor resulting in a radical. (b) Subsequent homolytic cleavage of the S–CF_3_ in the radical results in the formation of dibenzothiophene and ˙CF_3_.

In recent years, there has been a growing interest in employing dibenzothiophenium salts, like Umemoto's reagent, depicted in Scheme 7a, as electron acceptors in photoinduced reactions.^55^ Umemoto introduced the corresponding trifluoromethyl dibenzothiophenium salt in 1990 for trifluoromethylation applications with nucleophiles,^52–54^ such as aniline and triphenylphosphine. Roughly two decades afterward, Yasu, Koike and Akita demonstrated that Umemoto's reagent could act as ˙CF_3_ donor in the presence of [*fac*-Ir(ppy)_3_] as a photocatalyst. This is because the S–CF_3_ bond undergoes rapid homolytic cleavage upon reduction of Umemoto's reagent, as illustrated in Scheme 7b.^56^

In 2015, Yu and colleagues were the first to show that sulfonium salts could serve as electron acceptors in EDA complexes, specifically for trifluoromethylation reactions using Umemoto's reagent (Scheme 7a). By employing 4-methylmorpholine (NMM) as the electron donor, they successfully carried out the trifluoromethylation of a variety of substrates, including indoles and pyrroles, in good yields. The application of dibenzothiophenium salts as electron acceptors in EDAs has since widened, with notable examples including the work of Procter *et al.* on C–H alkylations and cyanations of arenes,^55^ as well as pentafluorocyclopropanation reactions introduced by Alcarazo.^56^ We will discuss the mechanistical studies reported by Yu *et al.* in their initial publication on the use of sulfonium salts in synthesis, contextualized with insights from more recent studies when needed. Yu *et al.* achieved the best results in DMF with acetonitrile as second best solvent choice. Given the broader availability of experimental data in acetonitrile compared to DMF, our discussion will concentrate on experiments conducted in acetonitrile as the solvent.

#### 4.3.1. Redox potentials and the EDA complex

**Fig. 14:**
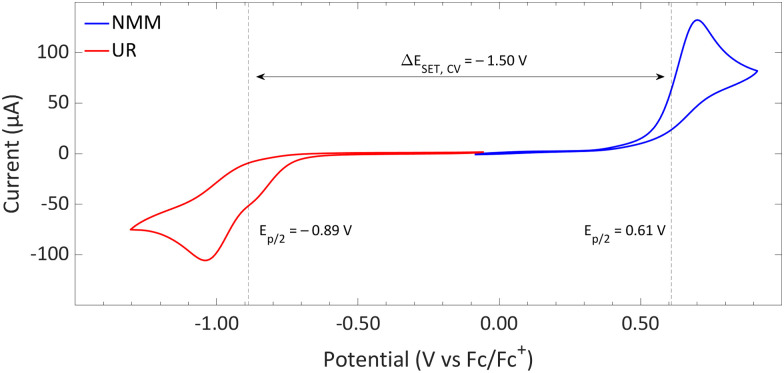
CVs of Umemoto's reagent (UR) and NMM in acetonitrile using [*n*Bu_4_N][BF_4_] (0.5 M) as supporting electrolyte at a scan rate of 100 mV s^−1^, showing irreversible redox events for both. Experimental details are reported in the ESI.†

To elucidate the characteristics of the SET process, an initial evaluation of the redox potentials in acetonitrile, as determined by cyclic voltammetry (Fig. 14), is essential.^57–60^ The analysis revealed half-peak potentials (*E*_p/2_) for Umemoto's reagent and NMM in acetonitrile of −0.89 V and 0.61 V, respectively. These findings lead to a highly endothermic Δ*E*_SET,CV_ of approximately −1.50 V, suggesting that the occurrence of a thermal SET is unlikely. Instead, a photoinduced SET could be feasible. Irreversible redox events were found for both the electron donor and acceptor, attributed to the decomposition of the radicals formed upon reduction or oxidation. Specifically, in its radical state, Umemoto's reagent demonstrates the ease of homolytic cleavage, resulting in the formation of ˙CF_3_. Similarly, the decomposition of NMM˙^+^ through a variety of radical disproportionation and recombination reactions is well-documented.^61^

**Fig. 15:**
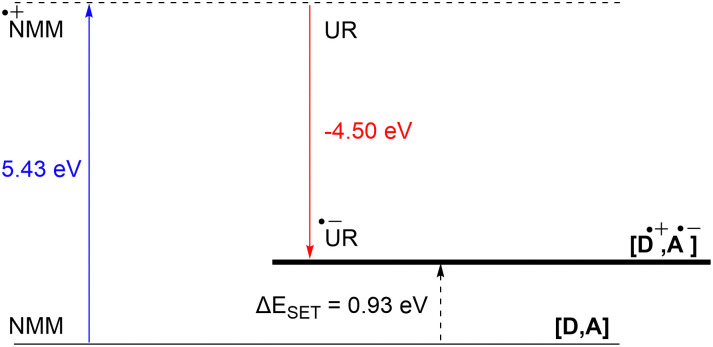
Energy diagram with calculated ionisation energy of NMM (blue) and electron affinities of Umemoto's reagent (UR) (red) and the estimated energy required for a SET in acetonitrile.

**Fig. 16:**
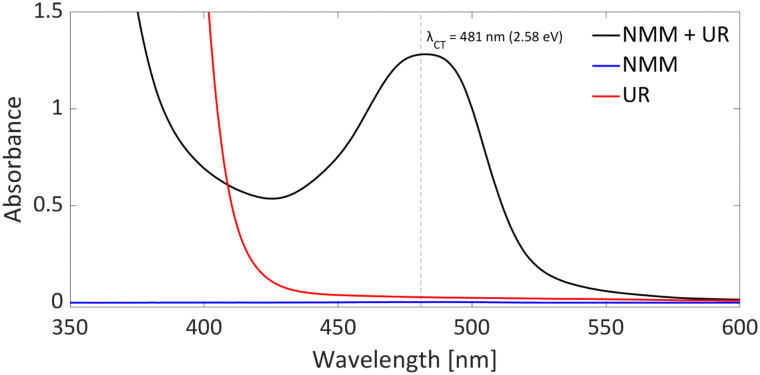
UV-vis spectrum of NMM (blue), Umemoto's reagent (UR) (red) and NMM + Umemoto's reagent (UR) (black) in acetonitrile with all concentrations being 11 mM.

DFT calculations conducted in acetonitrile reveal an IE for NMM of 5.43 eV and an EA for Umemoto's reagent of −4.50 eV. This leads to a Δ*E*_SET,calc_ of 0.93 eV (Fig. 15), underscoring the improbability of a thermal SET, while suggesting the potential for a photoinduced SET within the EDA complex [NMM, Umemoto's reagent]. Notably, while solutions of NMM and Umemoto's reagent individually appear colourless in acetonitrile, their combination results in a yellow solution. This colour change signifies the formation of the EDA complex [NMM, Umemoto's reagent], characterised by an absorbance band in the visible spectrum. The UV-vis spectroscopy analysis, depicted in Fig. 16, identifies the CT-band at 481 nm (2.58 eV), aligning with the yellow coloration. Application of the Benesi–Hildebrand method yielded a *K*_a_ of 18.7 M^−1^ in acetonitrile, indicating a markedly stronger interaction between the donor and acceptor in the EDA complex, compared to that between PMes_3_ and B(C_6_F_5_)_3_ (2.52 ± 0.43 M^−1^, discussed in Section 2.2). This enhanced interaction strength supports a greater mixing of orbitals between the donor and acceptor, thereby leading to a significant change in orbital energies and a substantial electronic coupling term (*ω*) in Mulliken theory. This provides a foundation for reconciling the differences between the Δ*E*_SET_ values obtained from CV measurements (−1.50 V), DFT calculations (0.93 eV), and the CT-band measured *via* UV-vis spectroscopy (2.58 eV).

#### 4.3.2. Characterisation of the radical pair

**Scheme 8:**
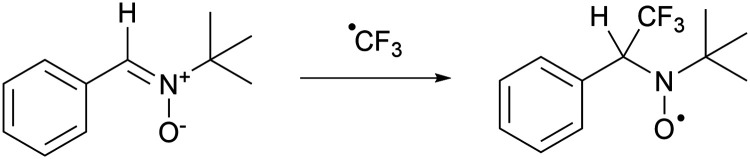
Trapping of CF_3_˙ with *tert*-butyl-α-phenylnitrone (PBN) results in a longer lived aminoxyl radical that can be observed by EPR spectroscopy.

**Fig. 17:**
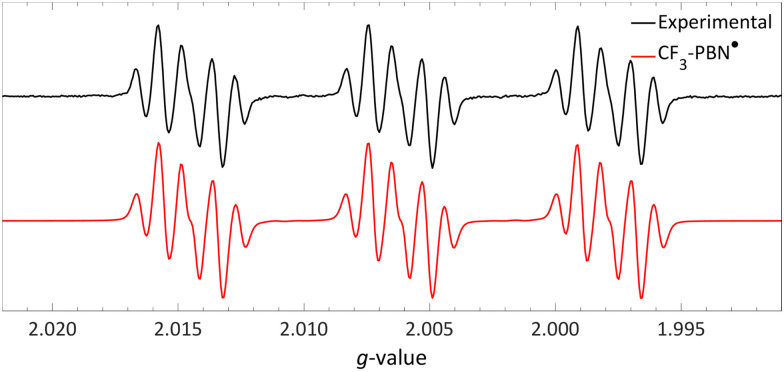
Room temperature EPR spectrum of the CF_3_–PBN˙ adduct (*g*_iso_ = 2.0061, ^19F^*a*_iso_ = 0.15 mT, ^14N^*a*_iso_ = 1.42 mT and ^1H^*a*_iso_ = 0.22 mT) after generation due to reduction of the sulfonium salt by NMM in acetonitrile. Further experimental and simulation details are reported in the ESI.†

After evaluating the nature of the SET process, we now focus on characterising the radical pair. The challenge here stems from the rapid decomposition of the sulfonium radical post-formation, which complicates its direct observation *via* EPR spectroscopy. To circumvent this issue, Yu and colleagues opted to indirectly establish the formation of the CF_3_˙ radical.^18^ They extended the lifetime of CF_3_˙, by trapping it with *N-tert*-butyl-α-phenylnitrone (PBN), forming a more stable aminoxyl radical (Scheme 8).^62^ This aminoxyl radical's presence was confirmed by EPR spectroscopy under the reaction conditions (room temperature and ambient light in acetonitrile; *g*_iso_ = 2.0061; Fig. 17). The EPR spectrum revealed hyperfine couplings with the three fluorine atoms of the CF_3_ group (3× ^19F^*a*_iso_ = 4.1 MHz, 0.15 mT), alongside the characteristic hyperfine couplings of a PBN radical (^14N^*a*_iso_ = 39.9 MHz, 1.42 mT and ^1H^*a*_iso_ = 6.0 MHz, 0.22 mT). Additional evidence for the *in situ* generation of CF_3_˙ was provided by the detection of the CF_3_–TEMPO adduct by ^19^F-NMR spectroscopy (*δ* = −55 ppm, 3.3% NMR yield), when the reaction was performed in the presence of TEMPO. Notably, introducing TEMPO not only facilitated the identification of the adduct but also completely suppressed the production of the trifluoromethylated target product.

Interestingly, the authors did not specifically address the detection of NMM˙^+^, leaving open the question of whether NMM serves as the exclusive electron donor in the reaction. In fact, in a related study Alcarazo *et al.* developed a perfluorocyclopropanation reaction using a sulfonium salt similar to Umemoto's reagent as electron acceptor without the need for NMM either as the electron donor or Brønsted base.^56^ Instead, NaHCO_3_ was utilized solely as a Brønsted base, and no separate electron donor was required. Alcarazo and colleagues suggested that the arene or heteroarene substrate, being moderately nucleophilic, fulfils the role of the electron donor, indicating that additional electron donors like NMM are not essential. This aligns with findings by Yu *et al.*, where the formation of the PBN-CF_3_˙ adduct was observed by EPR spectroscopy even without NMM, provided an indole substrate was present. Thus, it appears that both NMM and the indole substrate could serve as electron donors in Yu and colleagues’ reaction. The quantum yield for the trifluoromethylation was not determined by Yu *et al.* However, Alcarazo and colleagues reported a quantum yield of 3.4 for their perfluorocyclopropanation reaction using a sulfonium salt,^56^ suggesting a self-propagating radical chain mechanism. Such a mechanism is likely at play in Yu *et al.*'s trifluoromethylation as well. Furthermore, the reported quantum yield underscores the importance of a productive photoreaction that surpasses the BET, highlighting that for maximal efficiency, a chemical reaction of one of the radicals formed (in this case, the homolytic cleavage of the S–CF_3_ bond) should occur swiftly to prevent the BET from diminishing the reaction's productivity.

In summary, it is evident that the trifluoromethylation process using Umemoto's reagentin conjunction with NMM is initiated by a photoinduced SET towards Umemoto's reagent. Although NMM is currently posited as the electron donor, it is feasible that the substrate itself could also function as an effective electron donor. Given that this transformation is driven by photoinduction, the reaction's efficiency can benefit from targeted irradiation at the CT-band (*λ*_CT,max_ = 481 nm) associated with either the EDA complex [NMM, Umemoto's reagent] or [substrate, Umemoto's reagent]. This will likely shorten the current reaction time of 18 hours, and further elucidate which electron donor—NMM or the substrate—plays a pivotal role in the reaction mechanism.

## 5. Conclusions and perspective

This tutorial review demonstrated that the redox properties of the electron donor (Lewis base) and electron acceptor (Lewis acid) can be used to predict the feasibility of a thermal SET, as a radical pair should be no more than 0.4 eV higher in energy than the closed-shell state, hence, the Δ*E*_SET_ should be less than 0.4 eV. This can be determined experimentally, using cyclic voltammetry (CV) by comparing the associated redox potentials, or theoretically by calculating the ionisation energies and electron affinities. For the model system of PMes_3_ and [CPh_3_][B(C_6_F_5_)_4_] in DCM, CV showed a Δ*E*_SET,CV_ of −0.40 V, therefore supporting a thermal SET. Changing the trityl cation (CPh_3_^+^) to the weaker electron acceptor B(C_6_F_5_)_3_ results in a larger Δ*E*_SET,CV_ of −1.65 V, making a thermal SET inaccessible, while a photoinduced SET using visible light becomes feasible. Additionally, DFT calculations can be used to calculate the Δ*E*_SET,calc_ in various solvents and provide an estimation for the feasibility of a thermal or photoinduced SET. In agreement with the CV measurements, the obtained Δ*E*_SET,calc_ values predict a thermal SET for PMes_3_/CPh_3_^+^ (Δ*E*_SET,calc_ = 0.29 eV in toluene) and a photoinduced SET for PMes_3_/B(C_6_F_5_)_3_ (Δ*E*_SET,calc_ = 2.46 eV in toluene). To further investigate the EDA complexes associated with a photoinduced SET mechanism, time-dependent DFT (TD-DFT) calculations can be used to estimate the CT-band and identify the frontier molecular orbitals involved in the SET event. UV-vis spectroscopy can be used to determine the actual value of Δ*E*_SET_ for photoinduced SET, which serves as a guiding principle for the required wavelength to induce SET. Moreover, UV-vis titration of the CT-band can be used to determine the concentration and the association constant (*K*_a_) of the EDA complex.

For the characterisation of a radical pair, it is crucial to utilise specific conditions that enable the simultaneous observation of both formed radicals. In the case of a thermal SET, experiments should be conducted in the absence of light to eliminate the possibility of photoinduced SET processes. Feasible techniques for observing the pair of radicals include EPR and UV-vis spectroscopy. For a photoinduced SET event, it is necessary to confirm that radical formation does not occur prior to irradiation, to ascertain the photoinduced nature of the SET. Moreover, given the typically brief lifetime of these transient, high-energy species and the often rapid rate of back electron transfer (BET) at room temperature, techniques such as low-temperature EPR spectroscopy with *in situ* irradiation or short-pulsed transient absorption spectroscopy are essential for characterizing the radical pair. Additionally, resonance Raman spectroscopy can be instrumental in demonstrating that both species participate in the SET process.

The deployment of the discussed methods provides valuable mechanistic insights into the SET process, yet these approaches may not always be applicable to every system under study. For instance, in the context of C–H activation using the HMDS˙/TEMPO˙ radical pair, the authors were limited to detecting only the TEMPO˙ *via* EPR, owing to the fleeting existence of HMDS˙. The presence of HMDS˙ was inferred indirectly through methods such as radical trapping. Furthermore, the system TCNQ-{B(C_6_F_5_)_3_}_4_/(*p*BrPh)_3_N demonstrates how the redox properties of the electron acceptor, in this case TCNQ, can be modulated by incorporating Lewis acids like B(C_6_F_5_)_3_. Such redox tuning facilitates a thermal SET for this system, paving the way for the strategic selection of more apt electron donors and acceptors. In systems involving NMM/Umemoto's reagent, direct observation of the radicals was unattainable. The inference of a SET occurrence was supported by the identification of a trapped decomposition product of Umemoto's reagent. The precise identity of the electron donor in this situation remains ambiguous, leaving open the possibility of involvement from multiple electron donors.

We trust that this tutorial will assist in the accurate characterization of individual electron donors and acceptors, as well as the radical pairs, by showcasing the diverse methods that can be employed. Specifically, our goal is to foster a broad comprehension of the distinctions between thermal and photoinduced SET processes and the implications of these mechanisms for the spectroscopic techniques utilized. It should be noted, however, that some systems may not conform to the behaviours observed in the model systems discussed here, necessitating more sophisticated approaches to completely unravel the mechanisms underlying the formation of radical pairs.

## Author contributions

L. J. C. v. d. Z., J. H., J. M. v. G.: conceptualisation, reproduction of experimental and computational data from previously published work, and writing. J. C. S.: conceptualisation, supervision, writing, and funding acquisition.

## Conflicts of interest

There are no conflicts to declare.

## Note added after first publication

This article replaces the version published on 16 April 2024, where Fig. 2 was reproduced as Fig. 1.

## Supplementary Material

CS-053-D4CS00185K-s001
