# Supplementary material for: Mechanistic studies on single-electron transfer in frustrated Lewis pairs and its application to main-group chemistry
Source: Chem Soc Rev. 2024 Apr 16;53(10):4862–76. doi: 10.1039/d4cs00185k (PMC11104263; doi:10.1039/d4cs00185k)
Supplement: CS-053-D4CS00185K-s001 [file CS-053-D4CS00185K-s001.pdf]

Electronic Supplementary Material (ESI) for Chemical Society Reviews.
This journal is © The Royal Society of Chemistry 2024

Supporting Information

Mechanistic Studies on Single Electron Transfer in Frustrated Lewis Pairs and its Application to Main-Group Chemistry

Lars J. C. van der Zee,[a,†] *Jelle Hofman,*[a,†] *Joost M. van Gaalen*[a,†] *and J. Chris Slootweg*[a,*]

[a] Van 't Hoff Institute for Molecular Sciences, University of Amsterdam, PO Box 94157, 1090 GD Amsterdam, The Netherlands; e-mail: j.c.slootweg@uva.nl

† These authors contributed equally.

Table of Contents

I. Experimental Section....3
II. Electrochemistry....4
III. UV-vis and Transient Absorption Spectroscopy....13
IV. EPR Spectroscopy....15
V. Computational Details....18
VI. References....28

All experimental and computational data presented in the tutorial review and accompanying supporting information were generated by the authors. This effort aimed to replicate previously published work and offer readers a comprehensive summary of all essential data. Additionally, we created the images ourselves to enhance this resource.

I. Experimental Section

General Procedures. All manipulations were carried out under an atmosphere of purified nitrogen using standard Schlenk and glovebox techniques. Toluene was distilled from sodium. Dichloromethane (DCM) was distilled from CaH_2. Acetonitrile was obtained from a MBraun Solvent Purification System. $PhCF_3$ was distilled over P_2O_5. All solvents were stored over activated molecular sieves (4 Å) and degassed. $PMes_3$, $B(C_6F_5)_3$, $[CPh_3][B(C_6F_5)_4]$, Umemoto's reagent, N-Methylpyrrolidone (NMM), N-*tert*-Butyl-α-phenylnitrone (PBN), $(p\mathrm{BrPh})_3N$ and lithium bis(trimethylsilyl)amide (LiHMDS) were obtained from commercial sources and used as received, except NMM which was degassed before use. $[TEMPO][BF_4]$ was prepared according to literature procedure.[1] TCNQ was obtained from a commercial source and sublimated prior to use. The used electrolytes $[n\mathrm{Bu}_4N][PF_6]$ and $[n\mathrm{Bu}_4N][BF_4]$ were acquired from commercial sources, recrystallised, and dried in vacuo prior to use according to literature procedures.[2] $[n\mathrm{Bu}_4N][B(C_6F_5)_4]$ was prepared and recrystallised according to a literature procedure.[3]

II. Electrochemistry

General procedures. Cyclic voltammetry measurements were done using a standard three electrode set-up in a commercially available, one compartment Dr. Bob cell using an Ivium Vertex 5A potentiostat. Prior to use, the cell and bubbler were rinsed with water, ethanol and acetone or, when needed, cleaned using a potassium permanganate (1 mg/mL) and sulfuric acid (0.5 M) solution in water, followed by a hydrochloric acid (0.01 M) and peroxide (10%) solution in water. It was then dried in an oven at 130 °C for 4 hours, assembled while hot and purged with nitrogen for at least 30 minutes. Prior to use, the working electrodes were polished using a 0.05 μm alumina slurry (BASi PK4-polishing kit), washed with demineralized water, sonicated in demineralized water for several minutes and dried using compressed air. If insufficient for a clean polish, diamond polish was used as needed (15 μm, 3 μm and 1 μm), which was washed off using methanol and dried using compressed air.[4] It was then polished using alumina. To minimize the Ohmic resistance of the cell, the electrodes were placed as closely together as possible without touching. Dry solvents were syringe filtered prior to use to remove mol sieve dust. Measurements were generally performed at RT, starting at 0 V, going to negative potentials first at a scan rate of 100 mV/s and a step size of 1 mV. Three scans were taken per measurement and the second scan was used. Between each measurement the diffusion layer was refreshed by gently shaking the electrochemical cell. After the final measurement, internal standard was added to the cell, nitrogen was bubbled through for 15 minutes and a final scan was taken to calibrate the reference electrode. CVs were plotted using MATLAB in the IUPAC style.

Determination of Potentials

Half wave potentials were determined by adding the anodic and cathodic peak potential for reversible and quasi-reversible processes and dividing by two. Half peak potentials were determined by first determining the baseline corrected peak anodic or cathodic current according to literature procedures.[5] In short, this is done by taking the tangent to the baseline and subtracting this from the measured peak current, as shown in Figure S1. The half peak current is then taken as half of this value, after which the corresponding half peak potential can be determined.

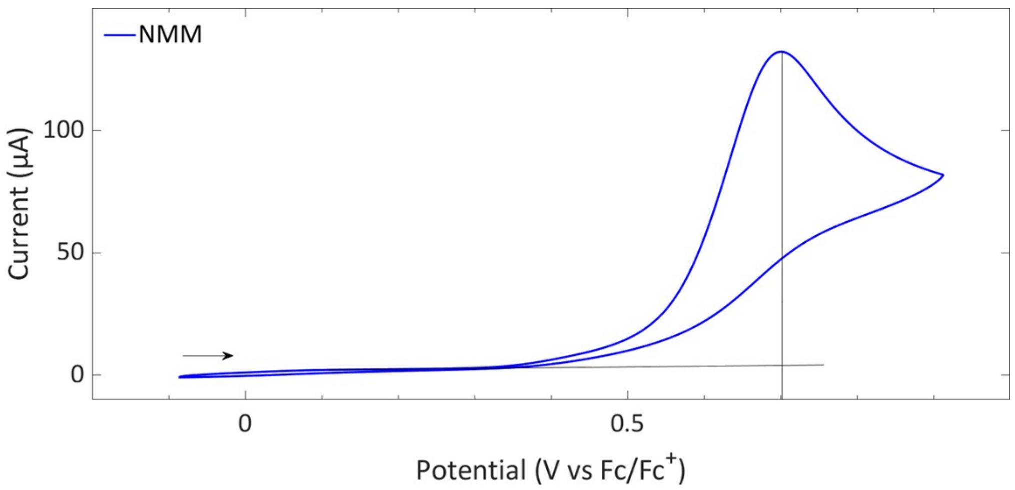

Figure S1 Determination of the baseline corrected peak current for NMM.

Cyclic Voltammetry: $PMes_3$

The procedure was adapted from literature.[6] In a glovebox, tris(2,4,6-trimethylphenyl)phosphine (19.3 mg for a 10 mM concentration for comparison with trityl cation or 19.4 mg, diluted 1:2, for a 5 mM concentration for comparison with $B(C_6F_5)_3$) and [$n$$Bu_4N$][$PF_6$] (973 mg) were dissolved in dry and degassed DCM (5 mL). This was added to the dr. Bob cell containing a Pt working electrode (Gamry, 3 mm diameter), Pt wire counter electrode (Thermo Scientific, 99.9% Pt, 0.25 mm diameter) and $Ag/AgNO_3$ reference electrode (10 mM $AgNO_3$, 0.1 M [$n$$Bu_4N$][$PF_6$] in MeCN). After the measurements, ferrocene was added as internal standard.

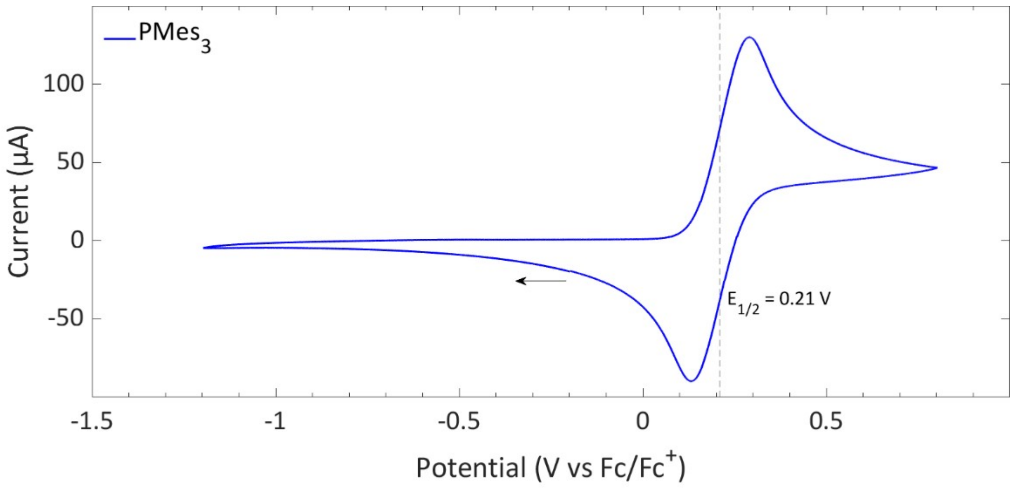

Figure S2 CV of $PMes_3$ (10 mM) in dry and degassed DCM with [$n$$Bu_4N$][$PF_6$] (0.5 M) as electrolyte. A Pt working electrode, Pt wire counter electrode and Ag wire reference electrode was used.

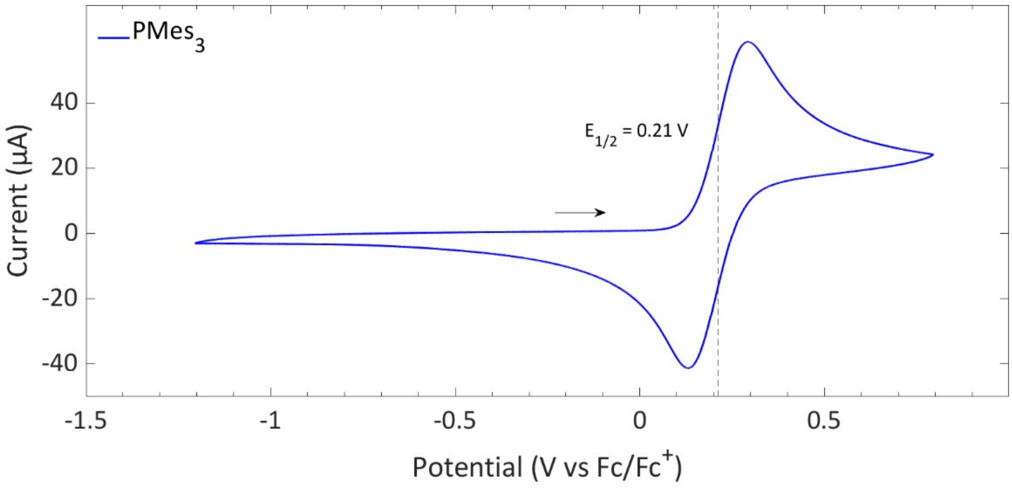

Figure S3 CV of $PMes_3$ (5 mM) in dry and degassed DCM with [nBu$_4$N][PF$_6$] (0.5 M) as electrolyte. A Pt working electrode, Pt wire counter electrode and Ag wire reference electrode was used.

Cyclic Voltammetry: [CPh_3][$B(C_6F_5)_4$]

The procedure was adapted from literature.[7] In a glovebox, triphenyl carbenium tetrakis(perfluorophenyl)borate (28.2 mg) and [nBu$_4$N][PF$_6$] (971 mg) were dissolved in dry and degassed DCM (5 mL). This was added to the dr. Bob cell containing a Pt working electrode (Gamry, 3 mm diameter), Pt wire counter electrode (Thermo Scientific, 99.9% Pt, 0.25 mm diameter) and Ag/$AgNO_3$ reference electrode (10 mM $AgNO_3$, 0.1 M [nBu$_4$N][PF$_6$] in MeCN). After the measurements, decamethylferrocene (97%, Sigma Aldrich) was added as internal standard due to overlap with the ferrocene redox couple. Decamethylferrocene was subsequently referenced to ferrocene via its literature redox potential of –0.59 V in DCM.[8]

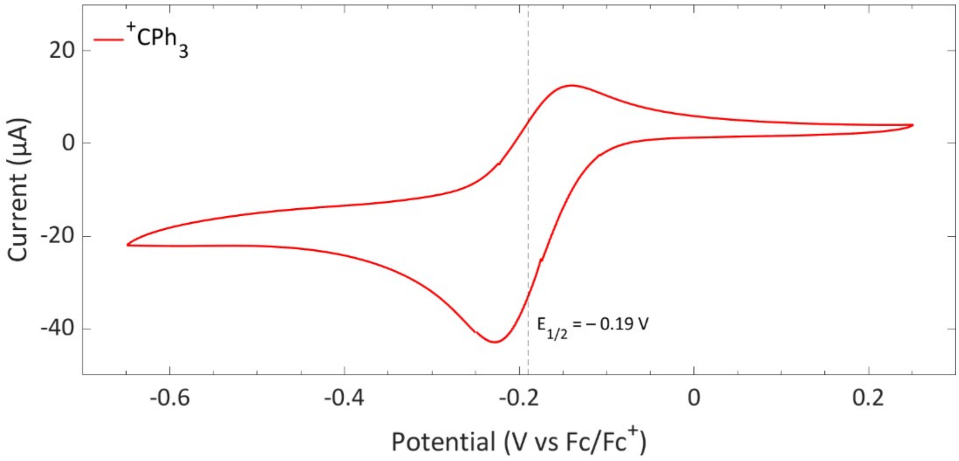

Figure S4 CV of triphenyl carbenium tetrakis(perfluorophenyl)borate (10 mM) in dry and degassed DCM with [nBu$_4$N][PF$_6$] (0.5 M) as electrolyte. A Pt working electrode, Pt wire counter electrode and Ag wire reference electrode was used.

Cyclic Voltammetry: $B(C_6F_5)_3$

The procedure was adapted from literature.[3] In a glovebox, tris(pentafluorophenyl)borate (12.8 mg) and tetra-*n*-butyl ammonium tetrakis (pentafluorophenyl)borate [nBu$_4$N][B(C_6F_5)$_4$] (234 mg) were dissolved in dry and degassed DCM (5 mL). This was added to the dr. Bob cell containing a Pt working electrode (Gamry, 3 mm diameter), Pt wire counter electrode (Thermo Scientific, 99.9% Pt, 0.25 mm diameter) and Ag wire reference electrode (STREM chemicals, 99.9%). The electrodes were left in solution for an hour prior to measuring to calibrate the reference electrode. After the measurements, ferrocene was added as internal standard.

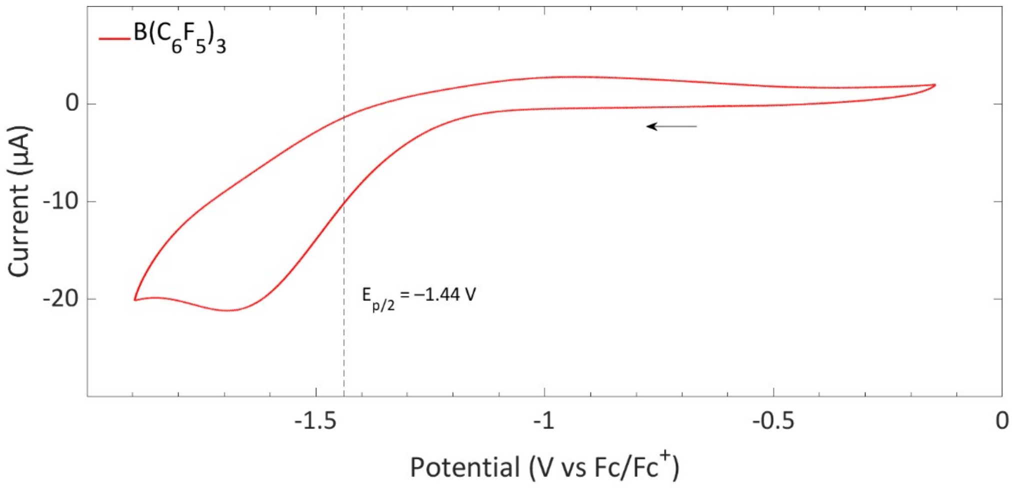

Figure S5 CV of tris(pentafluorophenyl)borane (5 mM) in dry and degassed DCM with [nBu$_4$N][B(C_6F_5)$_4$] (0.05 M) as electrolyte. A Pt working electrode, Pt wire counter electrode and Ag wire reference electrode was used.

Cyclic voltammetry: TEMPO

The procedure was adapted from literature.[9ref] In a glovebox TEMPO (15.6 mg) was dissolved in dry and degassed *ortho*-difluorobenzene (5 mL), diluted 1:10 and added to a dried dr. Bob cell containing [nBu$_4$N][PF_6] (390 mg). A glassy carbon (GC) working electrode (Gamry, 3 mm diameter), Pt wire counter electrode (Thermo Scientific, 99.9% Pt, 0.25 mm diameter) and Ag/$AgNO_3$ reference electrode (10 mM $AgNO_3$, 0.1 M [nBu$_4$N][PF_6] in MeCN) was used.

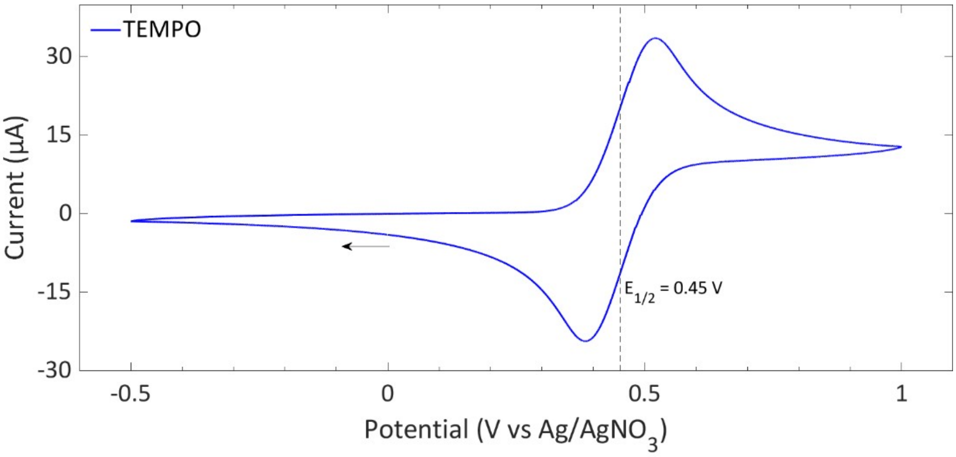

Figure S6 CV of TEMPO (2 mM) in dry and degassed *ortho*-difluorobenzene with [nBu$_4$N][PF$_6$] (0.2 M) as electrolyte. A GC working electrode, Pt wire counter electrode and Ag/AgNO$_3$ reference electrode was used.

Cyclic voltammetry: LiHMDS

The procedure was adapted from literature.[9] In a glovebox LiHMDS (16.8 mg) was dissolved in dry and degassed *ortho*-difluorobenzene (5 mL), diluted 1:10 and added to a dried dr. Bob cell containing [nBu$_4$N][PF$_6$] (390 mg). A GC working electrode (Metrohm, 3 mm diameter), Pt wire counter electrode (Thermo Scientific, 99.9% Pt, 0.25 mm diameter) and Ag/AgNO$_3$ reference electrode (10 mM AgNO$_3$, 0.1 M [nBu$_4$N][PF$_6$] in MeCN) was used.

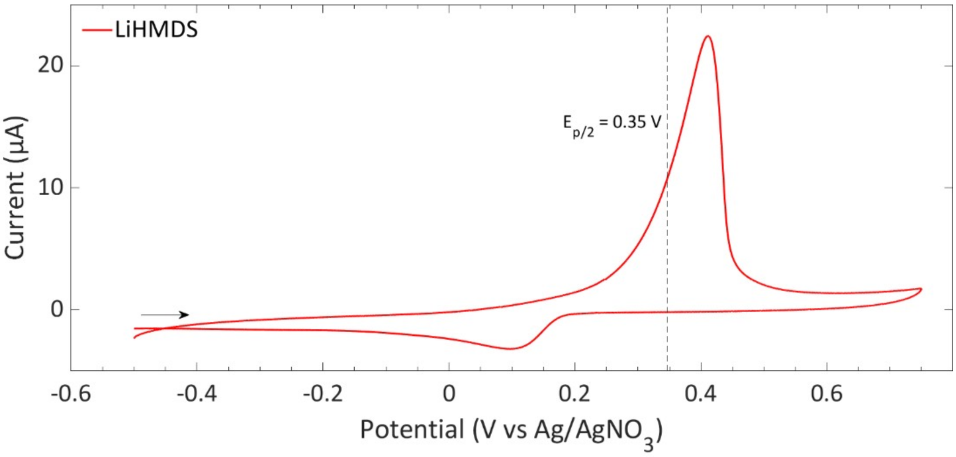

Figure S7 CV of LiHMDS (2 mM) in dry and degassed *ortho*-difluorobenzene with [nBu$_4$N][PF$_6$] (0.2 M) as electrolyte. A GC working electrode, Pt wire counter electrode and Ag/AgNO$_3$ reference electrode was used. Scan 2 and scan 3 do not fully overlap due to a change in current between scans.

Cyclic voltammetry: Ferrocene

Under a nitrogen atmosphere [nBu$_4$N][PF$_6$] (386 mg) was dissolved in dry and degassed *ortho*-difluorobenzene (5 mL). This was added to the dr. Bob cell, containing a few granules of

ferrocene. A GC working electrode (Metrohm, 3 mm diameter), Pt wire counter electrode (Thermo Scientific, 99.9% Pt, 0.25 mm diameter) and Ag/$AgNO_3$ reference electrode (10 mM $AgNO_3$, 0.1 M [*n*Bu_4N][PF_6] in MeCN) was used. A scan rate of 10 mV/s was used instead of 100 mV/s.

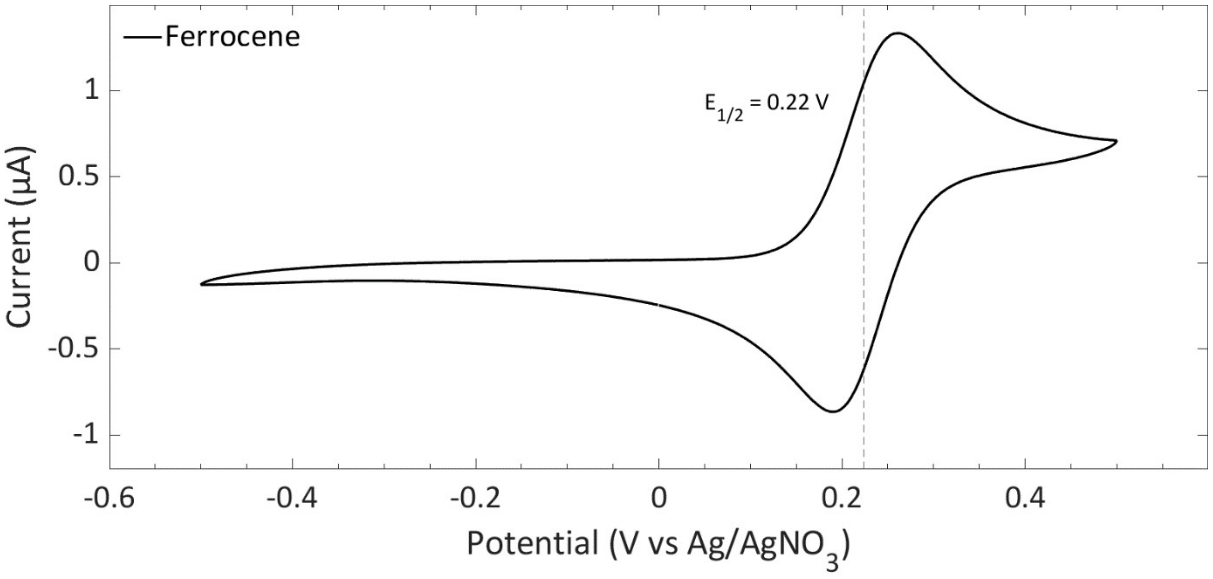

Figure S8 CV of ferrocene in dry and degassed *ortho*-difluorobenzene with [*n*Bu_4N][PF_6] (0.2 M) as electrolyte. A GC working electrode, Pt wire counter electrode and Ag/$AgNO_3$ reference electrode was used. A scan rate of 10 mV/s was used.

Cyclic voltammetry: N(*p*BrPh)$_3$

The procedure was adapted from literature.[8] In a glovebox tris(4-bromo-phenyl)amine (12.1 mg) was dissolved in dry and degassed DCM (5 mL), diluted 1:5 and added to a dried dr. Bob cell containing [*n*Bu_4N][PF_6] (960 mg). A Pt working electrode (Metrohm, 3 mm diameter), Pt wire counter electrode (Thermo Scientific, 99.9% Pt, 0.25 mm diameter) and Pt reference electrode was used. Prior to measuring the Pt reference electrode was allowed to equilibrate for an hour to prevent excessive drifting. After the measurements ferrocene was added as an internal standard and an additional measurement was taken.

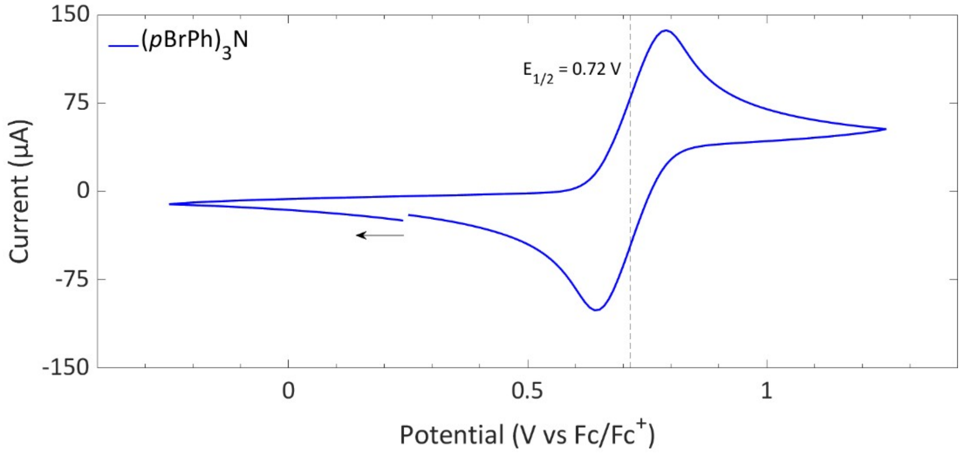

Figure S9 CV of N(*p*BrPh)$_3$ (10 mM) in dry and degassed DCM with [*n*Bu$_4$N][PF$_6$] (0.5 M) as electrolyte. A Pt working electrode, Pt wire counter electrode and Pt wire reference electrode was used. Scan 2 and scan 3 do not fully overlap due to a change in current between scans.

Cyclic voltammetry: TCNQ

The procedure was adapted from literature.[10] In a glovebox TCNQ (10.1 mg) was dissolved in dry and degassed DCM (10 mL) and added to a dried dr. Bob cell containing [*n*Bu$_4$N][PF$_6$] (387 mg). A Pt working electrode (Metrohm, 3 mm diameter), Pt wire counter electrode (Thermo Scientific, 99.9% Pt, 0.25 mm diameter) and Pt reference electrode was used. Prior to measuring the Pt reference electrode was allowed to equilibrate for an hour to prevent excessive drifting. After the measurements ferrocene was added as an internal standard and an additional measurement was taken.

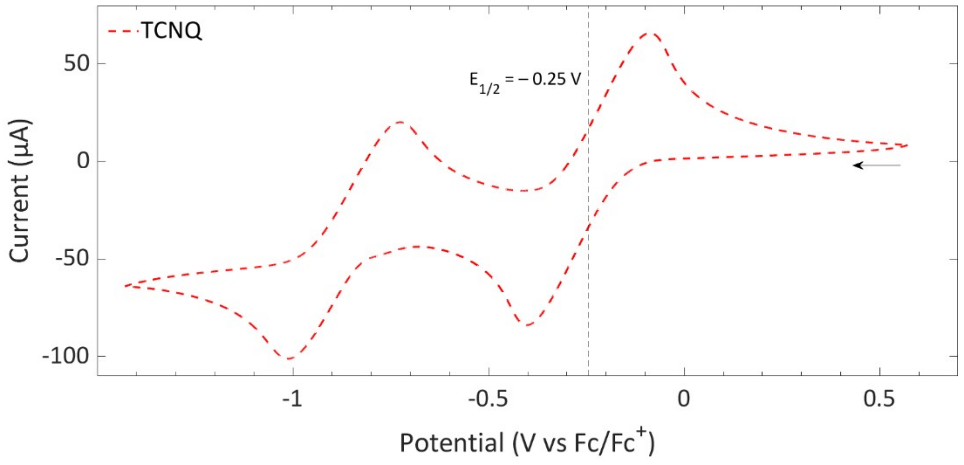

Figure S10 CV of TCNQ (5 mM) in dry and degassed DCM with [*n*Bu$_4$N][PF$_6$] (0.1 M) as electrolyte. A Pt working electrode, Pt wire counter electrode and Pt wire reference electrode was used.

Cyclic voltammetry: TCNQ-{B(C$_6$F$_5$)$_3$}$_4$

The procedure was adapted from literature.[11] In a glovebox TCNQ-{B(C$_6$F$_5$)$_3$}$_4$ was synthesized according to literature procedure. This was added directly to the Dr. Bob cell for

CV without further purification and without addition of electrolyte. A Pt working electrode (Metrohm, 3 mm diameter), Pt wire counter electrode (Thermo Scientific, 99.9% Pt, 0.25 mm diameter) and Pt reference electrode was used. Prior to measuring the Pt reference electrode was allowed to equilibrate for an hour to prevent excessive drifting. After the measurements ferrocene was added as an internal standard and an additional measurement was taken.

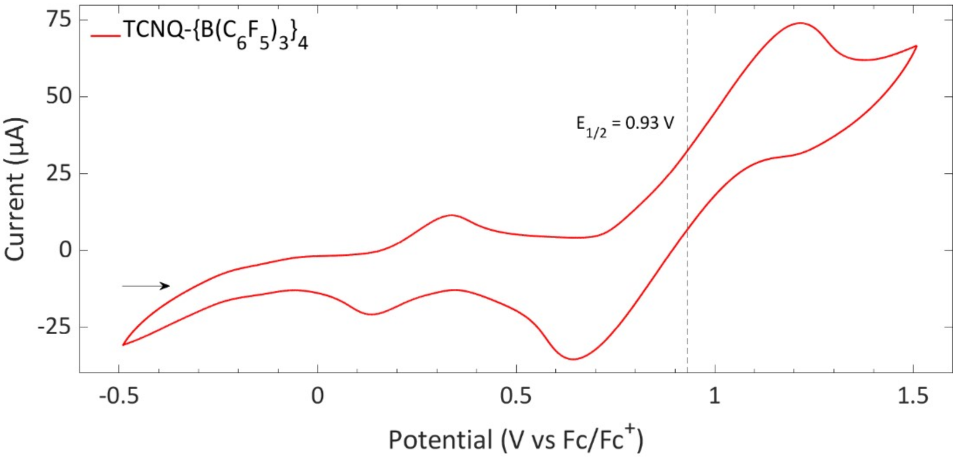

Figure S11 CV of TCNQ-$\{B(C_6F_5)_3\}_4$ (10 mM) in dry and degassed DCM without electrolyte. A Pt working electrode, Pt wire counter electrode and Pt wire reference electrode was used.

Cyclic Voltammetry: NMM

The procedure was adapted from literature.[12],[13] In a glovebox, *N*-methylmorpholine (53 mg) was dissolved in dry and degassed MeCN, diluted 1:10 and added to the dr. Bob cell containing [nBu$_4$N][BF$_4$] (853 mg). A Pt working electrode (Gamry, 3 mm diameter), Pt wire counter electrode (Thermo Scientific, 99.9% Pt, 0.25 mm diameter) and Ag/AgNO$_3$ reference electrode (10 mM AgNO$_3$, 0.1 M [nBu$_4$N][BF$_4$] in MeCN) was used. After the measurements, ferrocene was added as internal standard.

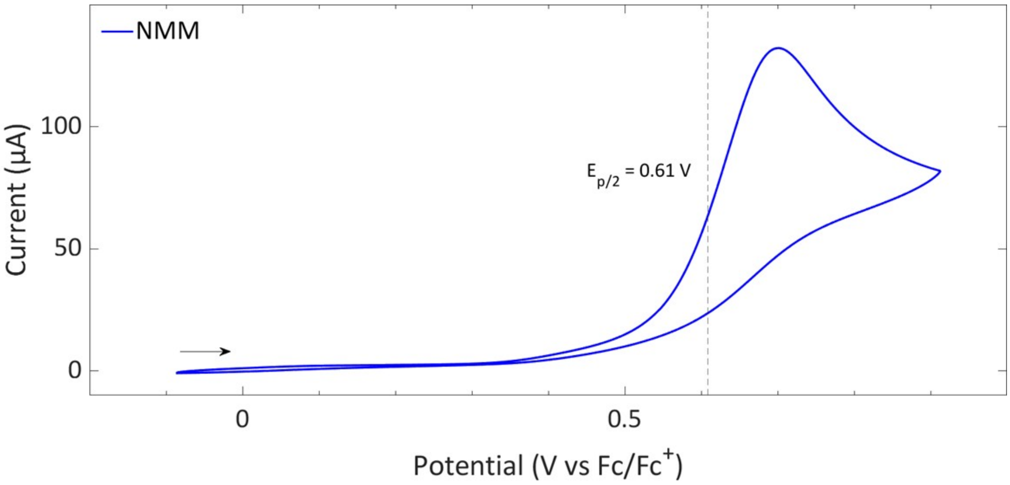

Figure S12 CV of N-methylmorpholine (10 mM) in dry and degassed MeCN with [nBu$_4$N][BF$_4$] (0.5 M) as electrolyte. A Pt working electrode, Pt wire counter electrode and AgNO$_3$ reference electrode was used.

Cyclic Voltammetry: Umemoto's Reagent

The procedure was adapted from literature.[14] In a glovebox, 5-(trifluoromethyl)dibenzothiophenium tetrafluoroborate (17 mg) was dissolved in dry and degassed MeCN and added to the dr. Bob cell with [nBu$_4$N][BF$_4$] (823 mg). A Pt working electrode (Gamry, 3 mm diameter), Pt wire counter electrode (Thermo Scientific, 99.9% Pt, 0.25 mm diameter) and Ag/AgNO$_3$ reference electrode (10 mM AgNO$_3$, 0.1 M [nBu$_4$N][BF$_4$] in MeCN) was used. After the measurements, ferrocene was added as internal standard.

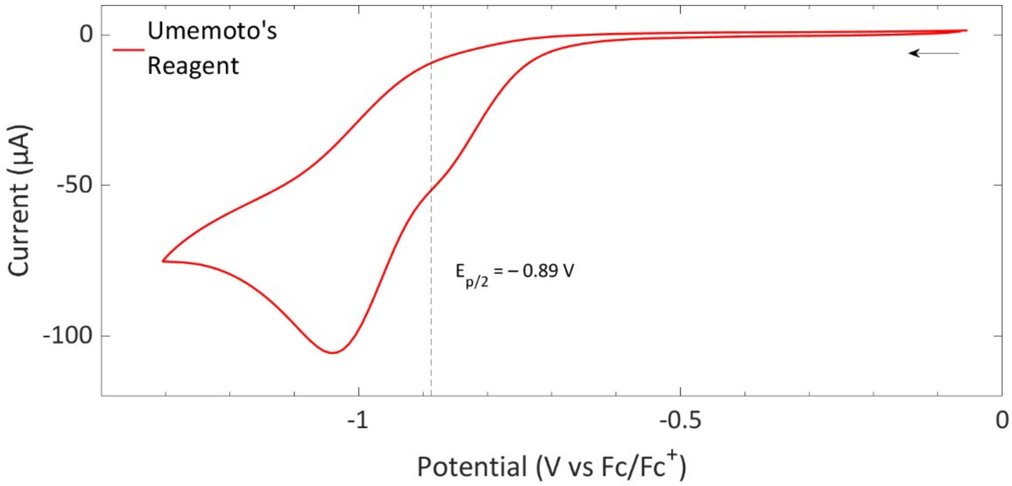

Figure S13 CV of 5-(trifluoromethyl)dibenzothiophenium tetrafluoro borate (10 mM) in dry and degassed MeCN with [nBu$_4$N][BF$_4$] (0.5 M) as electrolyte. A Pt working electrode, Pt wire counter electrode and AgNO$_3$ reference electrode was used.

III. UV-vis and Transient Absorption Spectroscopy

General procedures. The UV-vis spectroscopy data of $PMes_3$, $B(C_6F_5)_3$ and $PMes_3$ / $B(C_6F_5)_3$ and transient absorption spectroscopy of $PMes_3$ / $B(C_6F_5)_3$ were copied from reference 15. UV-vis spectra were recorded on a Shimadzu UV-2600i spectrophotometer in sealed cuvettes under nitrogen atmosphere.

Experimental results. $PMes_3$ / $B(C_6F_5)_3$ UV-vis Spectroscopy

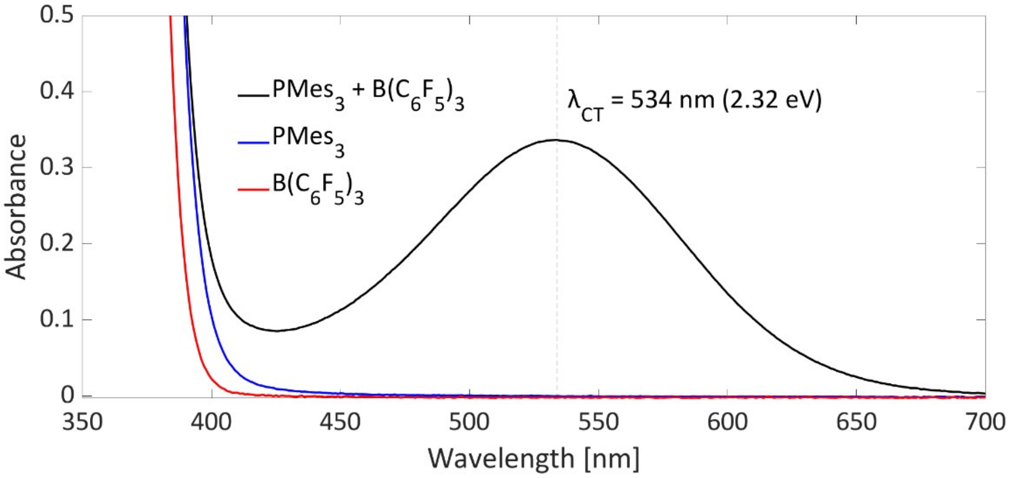

Figure S14 UV-vis spectra of toluene solutions of $PMes_3$ (45 mM), $B(C_6F_5)_3$ (30 mM) and the combination of $PMes_3$ (45 mM) and $B(C_6F_5)_3$ (30 mM).[15]

Experimental results. $PTipp_3$ / $[CPh_3][B(C_6F_5)_4]$ UV-vis Spectroscopy

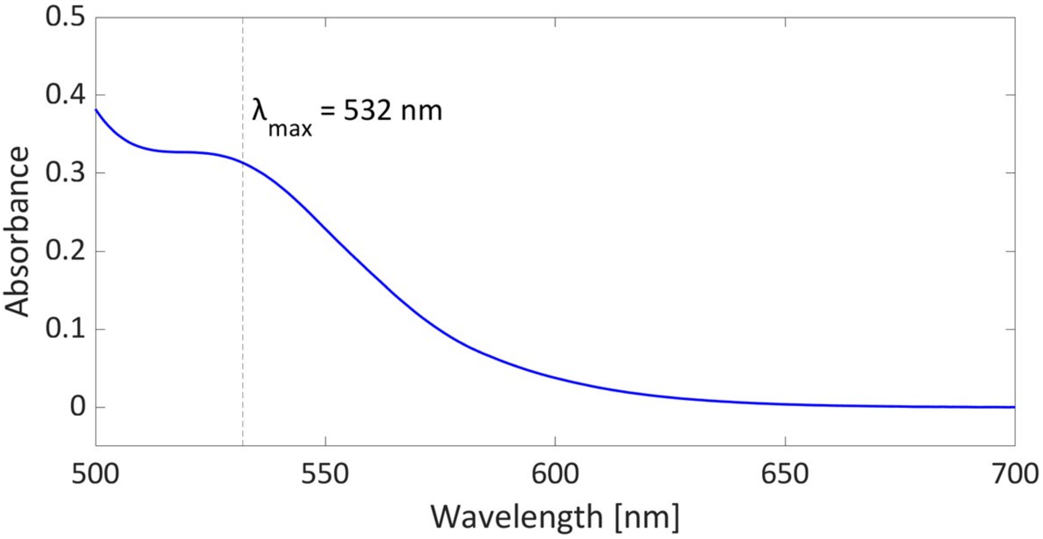

Figure S15 UV-vis spectra of a chlorobenzene solution of $PTipp_3$ (2.25 mM) and $[CPh_3][B(C_6F_5)_4]$ (2.25 mM).[16]

Experimental results. NMM / UR UV-vis Spectroscopy

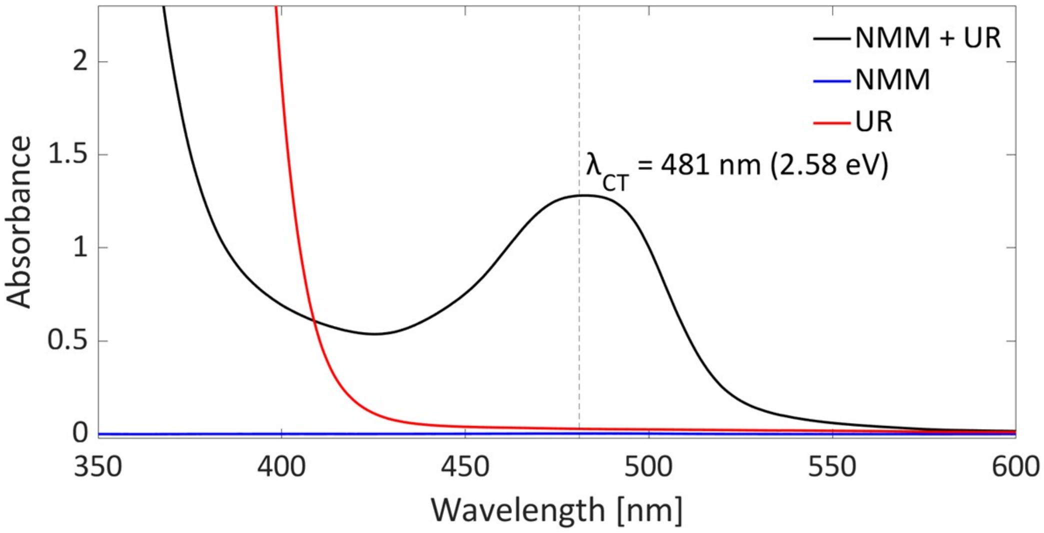

Figure S16: UV-vis spectra of acetonitrile solutions of NMM (11.25 mM) and UR (11.25 mM) and the combination thereof.[17]

Experimental results. $PMes_3$ / $B(C_6F_5)_3$ Transient Absorption Spectroscopy

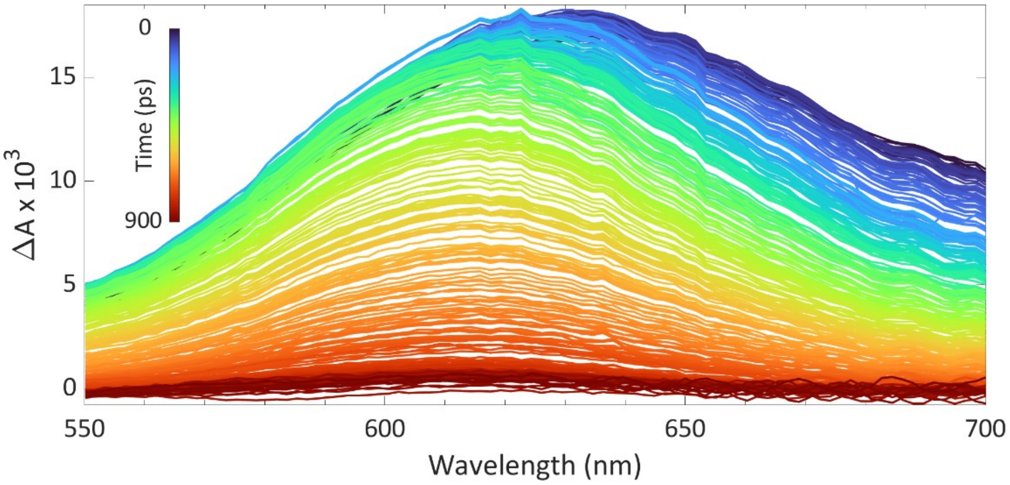

Figure S17: Transient Absorption Spectroscopy of a toluene solution of $PMes_3$ (40 mM) and $B(C_6F_5)_3$ (40 mM).[15]

IV. EPR Spectroscopy

General procedures. The experimental EPR spectroscopy data of $PMes_3$ / $[CPh_3][B(C_6F_5)_4]$ and $PMes_3$ / $B(C_6F_5)_3$ were copied from reference 15. All other EPR spectra were recorded on a Bruker EMXnano X-band spectrometer, equipped with a variable temperature controller. All the data was further analyzed and simulated using the MATLAB module EasySpin.[18]

Experimental results: $PMes_3$ / $[CPh_3][B(C_6F_5)_4]$

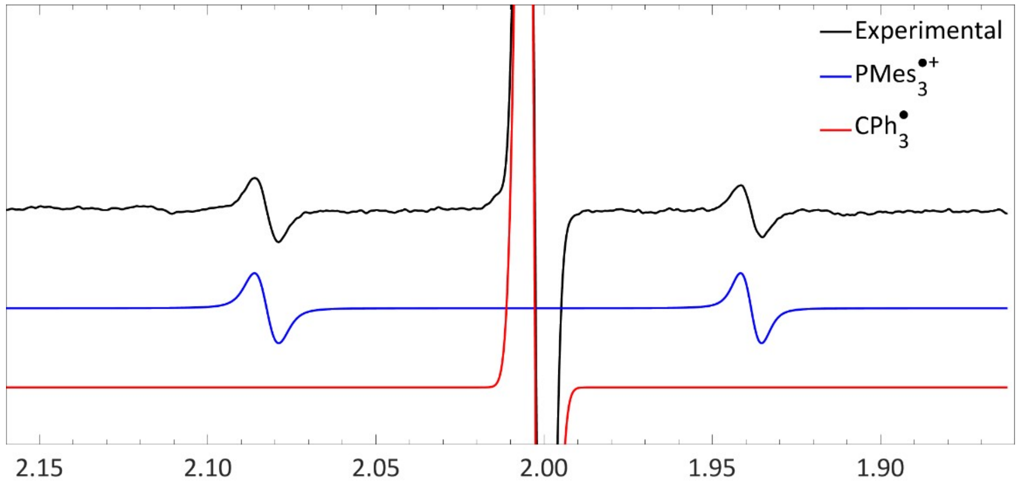

Figure S18: Room temperature EPR spectra of a solution of $PMes_3$ (60 mM) and $[CPh_3][B(C_6F_5)_4]$ (60 mM) in toluene.[15]

<u>Experimental details</u>: frequency: 9.381985 GHz; power: 0.6325 mW; Modulation: 4.000 G.
<u>Simulation details</u>: $PMes_3^{\bullet+}$: S = ½; g_{iso} = 2.0022; $^{31P}a_{iso}$ = 1149 MHz (41.0 mT); lwpp: 0.30 mT & 0.98 mT (Gaussian & Lorentzian). $CPh_3^{\bullet}$: S = ½; g_{iso} = 2.0027; lwpp: 0.99 mT (Gaussian). Ratio $PMes_3^{\bullet+}$: $CPh_3^{\bullet}$ = 1.0 : 6.8.

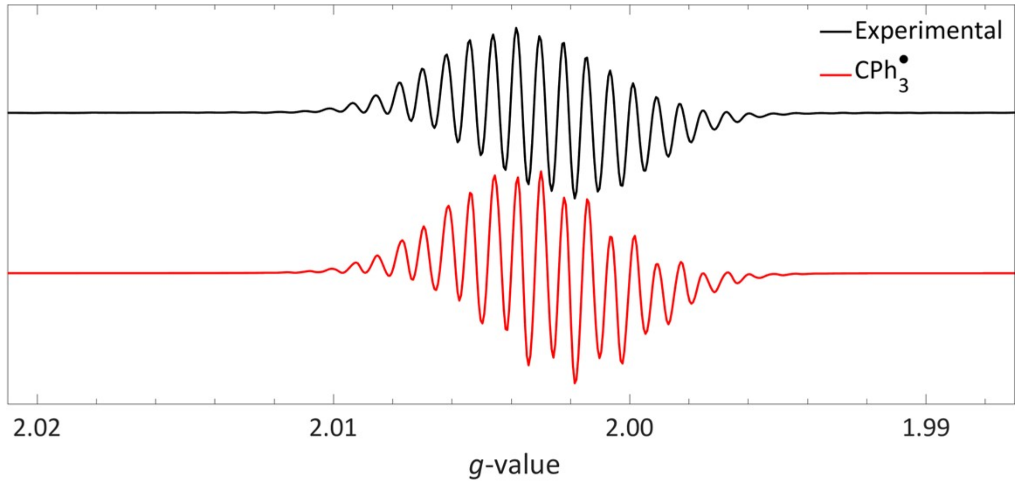

Figure S19: Room temperature EPR spectra of a solution of $PMes_3$ (60 mM) and $[CPh_3][B(C_6F_5)_4]$ (60 mM) in toluene.[15]

<u>Experimental details</u>: frequency: 9.381995 GHz; power: 0.6325 mW; Modulation: 1.000 G.

Simulation details: $CPh_3^{\bullet}$: S = ½; g_{iso} = 2.0028; 6 x $^{1H,o}a_{iso}$ = 7.29 MHz (0.260 mT); 6 x $^{1H,m}a_{iso}$ = 3.23 MHz (0.115 mT); 3 x $^{1H,p}a_{iso}$ = 7.85 MHz (0.280 mT); lwpp: 0.102 mT & 0.52 mT (Gaussian & Lorentzian).

Experimental results: $PMes_3$ / $B(C_6F_5)_3$

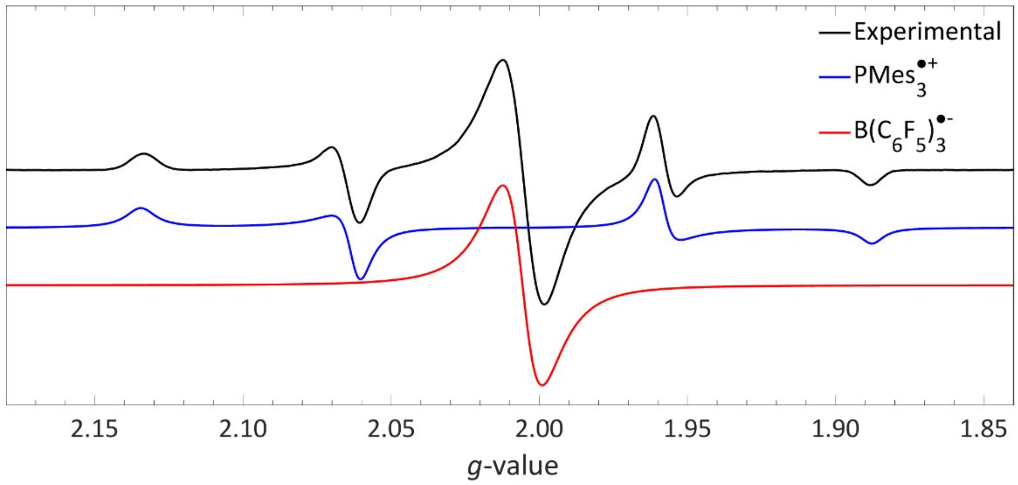

Figure S20: 30 K EPR spectra of a flash frozen glass of $PMes_3$ (60 mM) and $B(C_6F_5)_3$ (60 mM) in toluene using 390-500 nm irridiation.[15]

Experimental details: frequency: 9.375449 GHz; power: 0.6325 mW; Modulation: 2.000 G.

Simulation details: $PMes_3^{\bullet+}$: S = ½; $g_{\perp}$ = 2.0050; $g_{\parallel}$ = 2.0022; $^{31P}a_{\perp}$ = 477 MHz (17.0 mT); $^{31P}a_{\parallel}$ = 1149 MHz (41.0 mT); lwpp: 0.30 mT & 0.98 mT (Gaussian & Lorentzian). $B(C_6F_5)_3^{\bullet-}$: S = ½; g_{iso} = 2.0057; lwpp: 2.21 mT (Gaussian & Lorentzian). Ratio $PMes_3^{\bullet+}$: $B(C_6F_5)_3^{\bullet-}$ = 1.0 : 1.1.

Experimental results: LiHMDS / [TEMPO][BF_4]

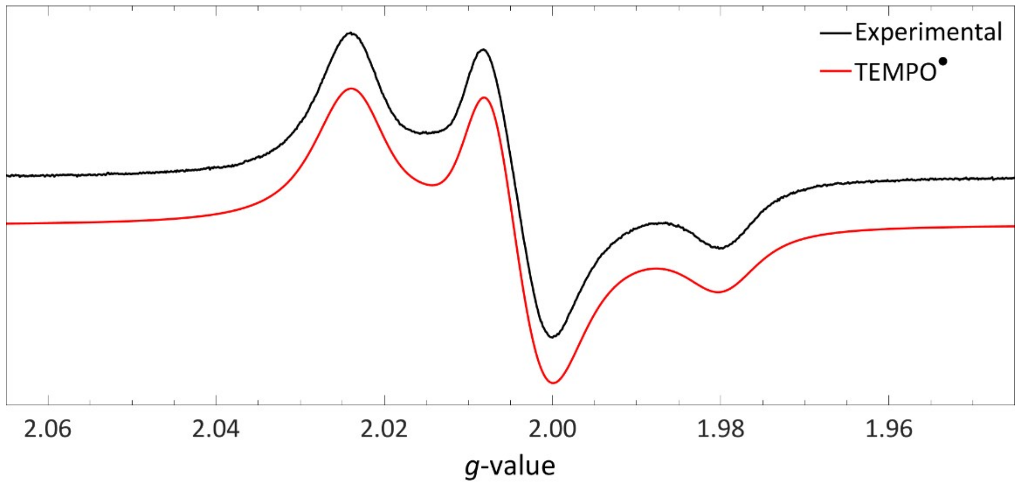

Figure S21: 100 K EPR spectra of a flash frozen solution of LiHMDS (5 mM) and [TEMPO][BF_4] (5 mM) in $PhCF_3$.[9]

Experimental details: frequency: 9.591344 GHz; power: 3.162 mW; Modulation: 2.000 G.

Simulation details: S = ½; $g_\perp$ = 2.0067; $g_\parallel$ = 2.0014; $^{14N}a_\perp$ = 21.5 MHz (0.77 mT); $^{14N}a_\parallel$ = 104 MHz (3.7 mT); lwpp: 0.62 mT & 0.87 mT (Gaussian & Lorentzian).

Experimental results: (*p*BrPh$_3$)N / TCNQ-{B(C$_6$F$_5$)$_3$}$_4$

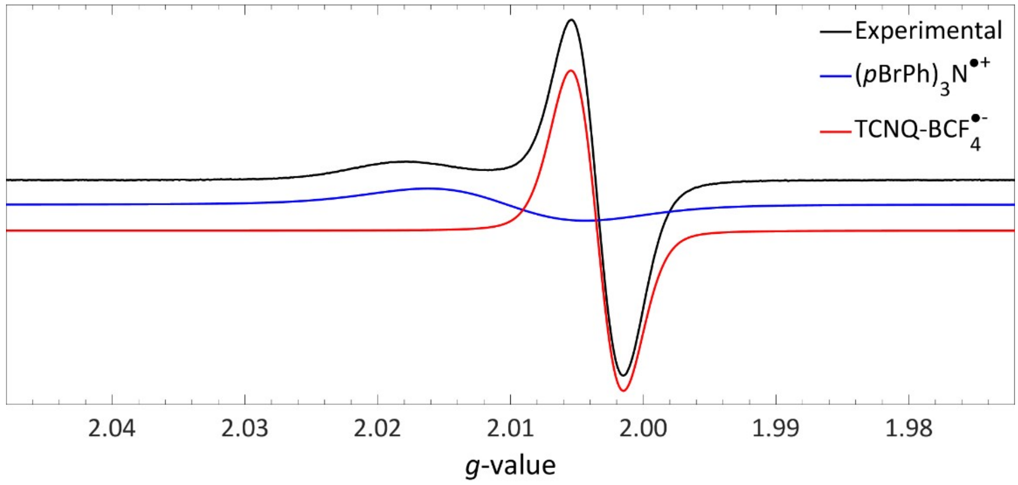

Figure S22: Room temperature EPR spectra of a solution of TCNQ (5 mM), (*p*BrPh)$_3$N (5 mM) and B(C$_6$F$_5$)$_3$ (20 mM) in DCM.[11]

Experimental details: frequency: 9.612491 GHz; power: 0.3162 mW; Modulation: 2.000 G.

Simulation details: (*p*BrPh$_3$)N$^{\bullet+}$: S = ½; g_{iso} = 2.0103; lwpp: 1.69 mT & 0.58 mT (Gaussian & Lorentzian). TCNQ-{B(C$_6$F$_5$)$_3$}$_4^{\bullet-}$: S = ½; g_{iso} = 2.0035; lwpp: 0.62 mT & 0.11 mT (Gaussian & Lorentzian). Ratio (*p*BrPh$_3$)N$^{\bullet+}$: TCNQ-{B(C$_6$F$_5$)$_3$}$_4^{\bullet-}$ = 1.00 : 0.97.

Experimental results: NMM / Umemoto's reagent

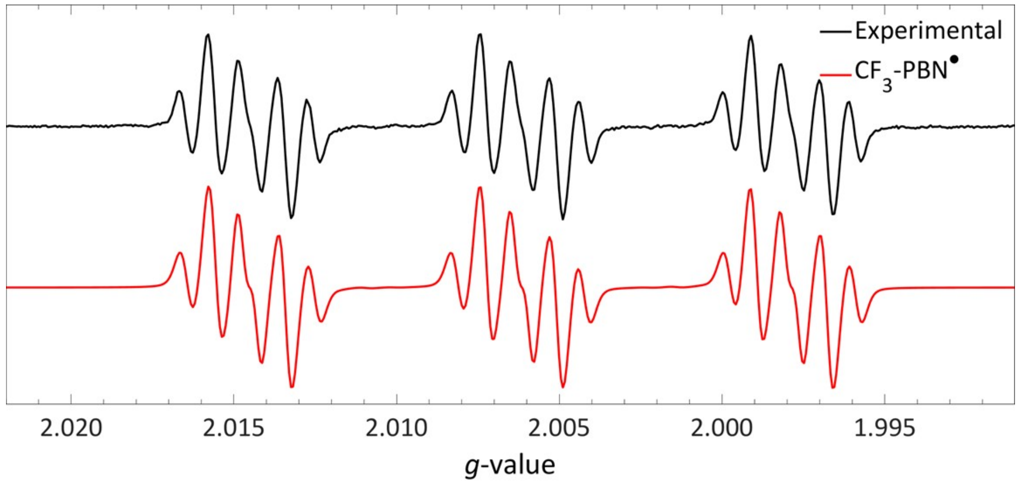

Figure 23: Room temperature EPR spectra of a solution of PBN (20 mM) and Umemoto's reagent (21 mM) and NMM (21 mM) in MeCN.[17]

Experimental details: frequency: 9.606383 GHz; power: 3.162 mW; Modulation: 1.000 G.

Simulation details: S = ½; g_{iso} = 2.0061; $^{14N}a_{iso}$ = 39.8 MHz (1.42 mT); $^{1H}a_{iso}$ = 6.1 MHz (0.22 mT); $^{19F}a_{iso}$ = 4.1 MHz (0.15 mT); lwpp: 0.063 mT & 0.211 mT (Gaussian & Lorentzian).

V. Computational Details

General procedures. All geometry optimizations were calculated using the (U)ωB97X-D density functional[19] and the 6-31G(d)[20,21] basis set as implemented in Gaussian16 (Revision C.01)[22] without symmetry constraints. The obtained geometries were characterized as true minima having no imaginary frequency. Single-point calculations on the optimized structures were performed using (U)ωB97X-D and the 6-311+G(d,p)[20,21] basis set taking solvents effects (Toluene, Chlorobenzene, DCM, Acetonitrile) into account by means of the self-consistent reaction field (SCRF) method using the polarizable continuum model (PCM).[23]

Calculated energies and ionisation energy and electron affinities

ΔE_{SET}	Gas phase	Toluene	Chlorobenzene	DCM	Acetonitrile
**$PMes_3$/ $B(C_6F_5)_3$**	3.96	2.47	1.91	1.77	1.59
**$PMes_3$/ CPh_3^+**	0.19	0.29	0.36	0.37	0.40
**NMM/ Umemoto reagent**	1.32	1.09	0.99	0.96	0.93
**(*p*BrPh)$_3$N/TCNQ**	3.30	1.65	1.00	0.83	0.61
**(*p*BrPh)$_3$N/ TCNQ_$(B(C_6F_5)_3)_4$**	0.62	-0.30	-0.57	-0.61	-0.64

Ionisation energies	Gas phase	Toluene	Chlorobenzene	DCM	Acetonitrile
**$PMes_3$**	6.27	5.50	5.23	5.16	5.07
**NMM**	7.46	6.23	5.73	5.60	5.43
**(*p*BrPh)$_3$N**	7.10	6.20	5.82	5.72	5.58

Electron Affinities	Gas phase	Toluene	Chlorobenzene	DCM	Acetonitrile
**$B(C_6F_5)_3$**	-2.31	-3.04	-3.31	-3.38	-3.47
**CPh_3^+**	-6.08	-5.21	-4.87	-4.78	-4.67
**Umemoto reagent**	-6.14	-5.14	-4.74	-4.64	-4.50
**TCNQ**	-3.80	-4.54	-4.82	-4.89	-4.97
**TCNQ_$(B(C_6F_5)_3)_4$**	-6.48	-6.49	-6.39	-6.33	-6.23

Cartesian coordinates (x,y,z) for the optimized geometries of the computed structures

$PMes_3$

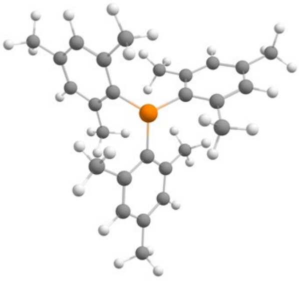

C	-1.540136000	-0.799684000	0.241530000
C	-1.649049000	-1.773241000	-0.775400000
C	-2.703494000	-0.433081000	0.962290000
C	-2.892186000	-2.360975000	-1.022910000
C	-3.924421000	-1.033617000	0.662240000
C	-4.039975000	-2.013479000	-0.319110000
H	-2.965125000	-3.107569000	-1.811410000
H	-4.810055000	-0.728483000	1.216110000
C	1.462515000	-0.934422000	0.241300000
C	2.360972000	-0.540344000	-0.774620000
C	1.726326000	-2.125905000	0.960470000
C	3.492104000	-1.322021000	-1.021600000
C	2.857556000	-2.882113000	0.660370000
C	3.764988000	-2.490479000	-0.318890000
H	4.175725000	-1.010932000	-1.809230000
H	3.035649000	-3.802277000	1.213300000
C	0.077403000	1.733151000	0.241930000
C	-0.712237000	2.314374000	-0.774410000
C	0.976825000	2.557233000	0.961900000
C	-0.600190000	3.684861000	-1.021400000
C	1.067130000	3.914980000	0.661690000

C	0.275455000	4.505163000	-0.318590000
H	-1.210568000	4.121715000	-1.809420000
H	1.774833000	4.529136000	1.215060000
P	-0.000029000	-0.000519000	0.860210000
C	0.789293000	-2.643076000	2.027150000
H	0.623190000	-1.895970000	2.810910000
H	-0.195440000	-2.892330000	1.614060000
H	1.195352000	-3.547129000	2.490240000
C	2.159440000	0.663092000	-1.665170000
H	1.140351000	0.713558000	-2.057800000
H	2.340735000	1.606625000	-1.141220000
H	2.844368000	0.615554000	-2.517020000
C	5.007800000	-3.296772000	-0.596430000
H	5.353441000	-3.157104000	-1.625530000
H	5.825525000	-2.994753000	0.069440000
H	4.833887000	-4.365844000	-0.437300000
C	-0.506494000	-2.199669000	-1.666700000
H	0.048186000	-1.342365000	-2.057380000
H	0.218448000	-2.831324000	-1.144240000
H	-0.890990000	-2.766453000	-2.519850000
C	-5.360241000	-2.685357000	-0.597430000
H	-5.508259000	-3.545321000	0.067270000
H	-6.198576000	-1.999637000	-0.437470000
H	-5.412345000	-3.053087000	-1.627040000
C	-2.681690000	0.636098000	2.029790000
H	-1.949741000	0.406657000	2.811800000
H	-2.406643000	1.613857000	1.616420000
H	-3.666888000	0.735260000	2.494700000
C	-1.653293000	1.538037000	-1.665400000
H	-1.186024000	0.631954000	-2.060510000
H	-2.560314000	1.220768000	-1.141690000
H	-1.956281000	2.156008000	-2.515810000
C	1.891962000	2.003748000	2.029220000
H	1.326814000	1.487668000	2.813000000
H	2.599200000	1.274229000	1.616830000
H	2.472779000	2.806926000	2.492060000
C	0.353524000	5.984355000	-0.597430000
H	0.069997000	6.211913000	-1.629830000
H	-0.324347000	6.541783000	0.060620000
H	1.364225000	6.369604000	-0.428610000

$PMes_3^{•+}$

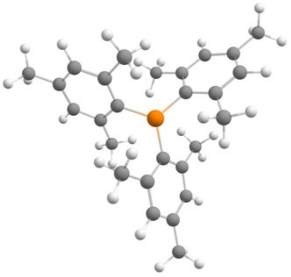

C	-0.351541000	1.724351000	0.126660000
C	-1.277385000	2.167427000	-0.850380000
C	0.331912000	2.648413000	0.956580000
C	-1.486914000	3.536724000	-0.973390000
C	0.082223000	4.003735000	0.780200000
C	-0.825317000	4.469583000	-0.172810000
H	-2.183395000	3.891482000	-1.728560000
H	0.603786000	4.720006000	1.409530000
C	-1.317648000	-1.166756000	0.126900000
C	-1.238470000	-2.189864000	-0.850390000
C	-2.459764000	-1.036973000	0.956830000
C	-2.319469000	-3.056041000	-0.973430000
C	-3.508638000	-1.930835000	0.780230000
C	-3.458233000	-2.949594000	-0.172900000
H	-2.278458000	-3.836409000	-1.728810000
H	-4.389805000	-1.837391000	1.409460000
C	1.669169000	-0.557885000	0.126810000
C	2.515235000	0.022095000	-0.850830000
C	2.128313000	-1.611549000	0.956970000
C	3.805944000	-0.480814000	-0.974110000
C	3.426936000	-2.072587000	0.780170000
C	4.283668000	-1.519718000	-0.173360000
H	4.461047000	-0.055007000	-1.729630000
H	3.787024000	-2.881994000	1.409720000
P	-0.000013000	-0.000115000	0.387300000
C	-2.580980000	0.039082000	2.008810000
H	-1.698021000	0.079488000	2.657590000
H	-2.704221000	1.031191000	1.560120000
H	-3.448305000	-0.145702000	2.646460000
C	-0.064798000	-2.365370000	-1.782890000
H	0.243385000	-1.420736000	-2.243360000
H	0.810213000	-2.773095000	-1.266300000
H	-0.325814000	-3.054536000	-2.589070000
C	-4.593454000	-3.925196000	-0.316910000
H	-4.666376000	-4.306713000	-1.338960000
H	-4.439231000	-4.784507000	0.346470000
H	-5.549921000	-3.468814000	-0.047850000
C	-2.017357000	1.239002000	-1.782180000
H	-1.355004000	0.496983000	-2.240460000
H	-2.810503000	0.688679000	-1.265650000
H	-2.480921000	1.809474000	-2.590060000
C	-1.102725000	5.940557000	-0.316180000
H	-1.921280000	6.237052000	0.350390000
H	-0.228015000	6.540460000	-0.050590000
H	-1.400537000	6.194407000	-1.337110000
C	1.324688000	2.214762000	2.008000000
H	0.919043000	1.428553000	2.655680000
H	2.245606000	1.826628000	1.558460000
H	1.597865000	3.057549000	2.646830000
C	2.080511000	1.126810000	-1.782700000
H	1.105306000	0.925117000	-2.238340000
H	2.002975000	2.089376000	-1.266780000
H	2.804665000	1.241464000	-2.592330000
C	1.257647000	-2.254812000	2.009380000
H	0.781477000	-1.511028000	2.659130000
H	0.459938000	-2.857634000	1.560970000
H	1.851837000	-2.913794000	2.646310000
C	5.696385000	-2.014524000	-0.317110000
H	6.064095000	-1.885050000	-1.338610000
H	6.362776000	-1.452417000	0.347890000
H	5.779347000	-3.071495000	-0.049840000

$B(C_6F_5)_3$

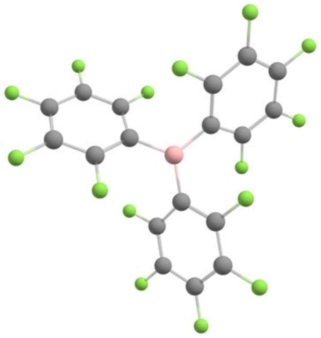

C	1.458890000	0.570374000	-0.000065000
C	2.489555000	-0.031103000	-0.727439000

C	3.784372000	0.465006000	-0.747002000
C	4.087809000	1.597588000	-0.000855000
C	3.097285000	2.224624000	0.745657000
C	1.809128000	1.711420000	0.726888000
F	2.245665000	-1.114156000	-1.468395000
F	4.731227000	-0.128606000	-1.466172000
F	5.321037000	2.079434000	-0.001219000
F	3.391230000	3.303108000	1.464382000
F	0.895866000	2.342310000	1.468132000
B	-0.000096000	0.000168000	0.000405000
C	-1.223410000	0.978559000	0.000338000
C	-1.217491000	2.172935000	-0.725341000
C	-2.294746000	3.046012000	-0.744708000
C	-3.428052000	2.741174000	-0.000239000
C	-3.476232000	1.568771000	0.744542000
C	-2.387410000	0.710188000	0.725773000
F	-0.157080000	2.504627000	-1.464895000
F	-2.253540000	4.163835000	-1.462245000
F	-4.462190000	3.567969000	-0.000582000
F	-4.557862000	1.282590000	1.461661000
F	-2.477773000	-0.397138000	1.465427000
C	-0.235544000	-1.548498000	0.000594000
C	-1.272590000	-2.140770000	-0.725270000
C	-1.490112000	-3.510216000	-0.744371000
C	-0.659646000	-4.339062000	0.000552000
C	0.379527000	-3.794370000	0.745505000
C	0.578739000	-2.422131000	0.726430000
F	-2.089836000	-1.388324000	-1.465131000
F	-2.478652000	-4.033602000	-1.461973000
F	-0.858558000	-5.648053000	0.000559000
F	1.167853000	-4.587833000	1.463175000
F	1.582617000	-1.946588000	1.466393000

$B(C_6F_5)_3^{\bullet-}$

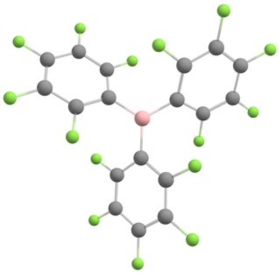

C	1.377806000	-0.750193000	-0.000148000
C	1.584926000	-1.975638000	-0.652741000
C	2.792897000	-2.655911000	-0.658455000
C	3.889677000	-2.117559000	0.000747000
C	3.746086000	-0.903990000	0.659426000
C	2.519069000	-0.258745000	0.652885000
F	0.588049000	-2.546846000	-1.348767000
F	2.921647000	-3.818983000	-1.313190000
F	5.066737000	-2.758257000	0.001271000
F	4.792419000	-0.380575000	1.314572000
F	2.457356000	0.888830000	1.348398000
B	-0.000112000	-0.000114000	-0.000292000
C	-0.039262000	1.568225000	-0.000247000
C	0.918843000	2.360028000	-0.652592000
C	0.904174000	3.746285000	-0.658627000
C	-0.110681000	4.427309000	-0.000220000
C	-1.090281000	3.696487000	0.658251000
C	-1.035724000	2.311218000	0.652076000
F	1.912224000	1.782100000	-1.348050000
F	1.847500000	4.439000000	-1.313044000
F	-0.144087000	5.767034000	-0.000348000
F	-2.066976000	4.341203000	1.312771000
F	-1.999048000	1.684286000	1.347386000
C	-1.338667000	-0.818292000	-0.000188000
C	-2.503497000	-0.384372000	-0.652399000
C	-3.696675000	-1.090251000	-0.658641000
C	-3.778947000	-2.309620000	-0.000099000
C	-2.656220000	-2.792562000	0.658355000
C	-1.483753000	-2.052790000	0.652076000
F	-2.499626000	0.765187000	-1.347340000
F	-4.768211000	-0.619767000	-1.313228000
F	-4.922420000	-3.008499000	0.000041000
F	-2.726345000	-3.960721000	1.312956000
F	-0.459004000	-2.573683000	1.347093000

CPh_3^+

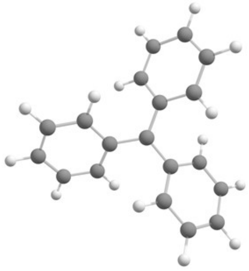

C	4.191182000	-0.630376000	-0.000215000
C	3.358768000	-1.517114000	0.685133000
C	1.987790000	-1.324540000	0.675537000
C	1.428641000	-0.215053000	-0.000029000
C	0.000144000	-0.000197000	-0.000110000
C	-0.527967000	1.344394000	-0.000177000
C	-1.733966000	1.641952000	-0.676190000
C	-2.227719000	2.935367000	-0.685682000
C	-1.549461000	3.944523000	0.000254000
C	-0.365560000	3.666700000	0.685903000
C	0.153128000	2.383094000	0.675962000
C	-0.900355000	-1.129701000	-0.000047000
C	-0.554765000	-2.323499000	-0.674893000
C	-1.428350000	-3.397511000	-0.684436000
C	-2.642081000	-3.313812000	0.000239000
C	-2.993760000	-2.149037000	0.684777000
C	-2.141067000	-1.058365000	0.674925000
C	2.289248000	0.681027000	-0.675613000
C	3.656242000	0.461977000	-0.685432000
H	5.264579000	-0.791742000	-0.000267000
H	3.783994000	-2.353468000	1.229126000
H	1.341632000	-1.995362000	1.231680000
H	-2.243314000	0.862341000	-1.232650000
H	-3.137677000	3.163289000	-1.229983000
H	-1.946409000	4.954801000	0.000472000
H	0.145976000	4.452917000	1.230370000
H	1.057014000	2.158707000	1.232279000
H	0.375540000	-2.375657000	-1.230458000
H	-1.170561000	-4.300002000	-1.227858000
H	-3.318855000	-4.162461000	0.000319000
H	-3.930937000	-2.098450000	1.228250000
H	-2.398664000	-0.162619000	1.230073000
H	1.868618000	1.512317000	-1.231382000
H	4.308510000	1.136408000	-1.229412000

$CPh_3^{\bullet}$

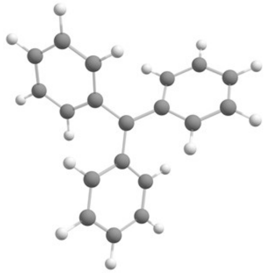

C	3.453014000	-2.536551000	-0.001033000
C	3.475316000	-1.317588000	-0.676799000
C	2.357999000	-0.493653000	-0.675823000
C	1.178215000	-0.866218000	-0.000406000
C	-0.000261000	-0.000514000	-0.000226000
C	-1.339389000	-0.587670000	0.000119000
C	-2.409269000	0.034245000	0.675010000
C	-3.679650000	-0.525572000	0.676164000
C	-3.924165000	-1.720367000	0.001075000
C	-2.880403000	-2.350536000	-0.674560000
C	-1.607727000	-1.795913000	-0.674392000
C	0.160612000	1.452974000	-0.000093000
C	1.232432000	2.068971000	0.677043000
C	1.382659000	3.449085000	0.677979000
C	0.471685000	4.257837000	0.000429000
C	-0.594304000	3.668564000	-0.677404000
C	-0.750162000	2.289048000	-0.677004000
C	1.176239000	-2.103564000	0.674783000
C	2.296912000	-2.922946000	0.675099000
H	4.328287000	-3.179239000	-0.001259000
H	4.367184000	-1.010115000	-1.215240000
H	2.382420000	0.446426000	-1.218249000
H	-2.227328000	0.957374000	1.216633000
H	-4.482840000	-0.030338000	1.214213000
H	-4.918743000	-2.156220000	0.001455000
H	-3.061025000	-3.276815000	-1.212384000
H	-0.806435000	-2.287978000	-1.216944000
H	1.939680000	1.450413000	1.220907000
H	2.211842000	3.897352000	1.217800000
H	0.591366000	5.337108000	0.000595000
H	-1.305020000	4.287830000	-1.217113000
H	-1.575679000	1.840896000	-1.221312000
H	0.286957000	-2.408449000	1.217734000
H	2.270806000	-3.866041000	1.213402000

NMM

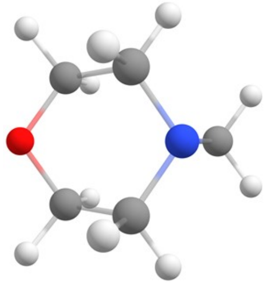

C	-0.334672000	0.965503000	1.167766000
O	-0.972487000	1.445850000	0.000000000
C	-0.334672000	0.965503000	-1.167766000
C	-0.334672000	-0.563971000	-1.192819000
N	0.270097000	-1.151572000	0.000000000
C	-0.334672000	-0.563971000	1.192819000
C	1.722335000	-1.115790000	0.000000000
H	-0.893030000	1.370879000	2.016494000
H	0.696515000	1.355076000	1.219691000
H	-0.893030000	1.370879000	-2.016494000
H	0.696515000	1.355076000	-1.219691000
H	-1.376063000	-0.907724000	-1.247533000
H	0.187483000	-0.935014000	-2.082457000
H	-1.376063000	-0.907724000	1.247533000
H	0.187483000	-0.935014000	2.082457000
H	2.092956000	-1.644511000	-0.884898000
H	2.092956000	-1.644511000	0.884898000
H	2.171602000	-0.106850000	0.000000000

$NMM^{\bullet +}$

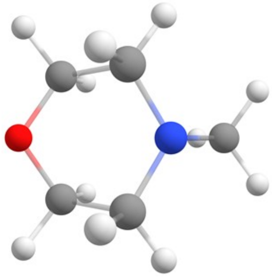

C	-0.329792000	1.035563000	1.162067000
O	-0.971943000	1.446802000	0.000000000
C	-0.329792000	1.035563000	-1.162067000
C	-0.329792000	-0.542210000	-1.207980000
N	0.296428000	-0.990163000	0.000000000
C	-0.329792000	-0.542210000	1.207980000
C	1.694111000	-1.372265000	0.000000000
H	-0.896864000	1.409387000	2.015093000
H	0.707284000	1.395106000	1.209898000
H	-0.896864000	1.409387000	-2.015093000
H	0.707284000	1.395106000	-1.209898000
H	-1.371510000	-0.874073000	-1.229380000
H	0.208660000	-0.903552000	-2.084958000
H	-1.371510000	-0.874073000	1.229380000
H	0.208660000	-0.903552000	2.084958000
H	1.915642000	-1.957081000	-0.894010000
H	1.915642000	-1.957081000	0.894010000
H	2.324473000	-0.469492000	0.000000000

Umemoto's reagent$^+$

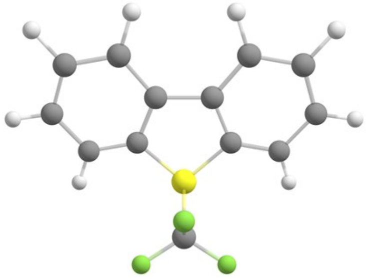

C	-0.937272000	0.199631000	3.499575000
C	-2.121475000	-0.329886000	2.985603000
C	-2.336174000	-0.434177000	1.611693000
C	-1.344956000	-0.008405000	0.734514000
C	-0.167308000	0.516215000	1.284009000
C	0.069198000	0.643073000	2.643268000
C	-1.344956000	-0.008405000	-0.734514000
C	-0.167308000	0.516215000	-1.284009000
S	0.961648000	1.013650000	0.000000000
C	-2.336174000	-0.434177000	-1.611693000
C	-2.121475000	-0.329886000	-2.985603000
C	-0.937272000	0.199631000	-3.499575000
C	0.069198000	0.643073000	-2.643268000

C	2.159798000	-0.450091000	0.000000000
F	2.894632000	-0.331137000	1.085879000
F	2.894632000	-0.331137000	-1.085879000
F	1.505426000	-1.585308000	0.000000000
H	-0.797039000	0.275830000	4.571990000
H	-2.895454000	-0.662817000	3.668882000
H	-3.267982000	-0.841985000	1.234404000
H	0.989714000	1.065460000	3.031303000
H	-3.267982000	-0.841985000	-1.234404000
H	-2.895454000	-0.662817000	-3.668882000
H	-0.797039000	0.275830000	-4.571990000
H	0.989714000	1.065460000	-3.031303000

Umemoto's reagent•

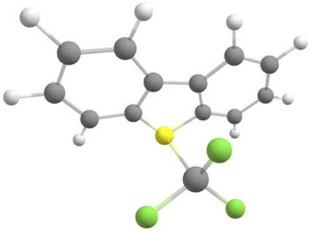

C	-3.519649000	0.726066000	-0.188333000
C	-3.009202000	0.589878000	-1.485385000
C	-1.641602000	0.616814000	-1.709744000
C	-0.766706000	0.783166000	-0.631399000
C	-1.294421000	0.915149000	0.664753000
C	-2.669159000	0.888679000	0.896251000
C	0.688588000	0.820563000	-0.633511000
C	1.212653000	0.979989000	0.661209000
S	-0.042799000	1.087882000	1.886208000
C	1.567892000	0.697150000	-1.714096000
C	2.935779000	0.737813000	-1.493197000
C	3.442228000	0.900127000	-0.197627000
C	2.587526000	1.021673000	0.889051000
C	0.105845000	-2.136992000	0.200198000
F	-0.917559000	-2.616496000	0.883465000
F	1.253259000	-2.546328000	0.709896000
F	0.014072000	-2.456967000	-1.079097000
H	-4.592892000	0.701250000	-0.026464000
H	-3.690258000	0.459718000	-2.320539000
H	-1.249256000	0.505378000	-2.716381000
H	-3.065970000	0.989036000	1.901539000
H	1.178957000	0.565510000	-2.719615000
H	3.620286000	0.640588000	-2.330010000
H	4.515792000	0.928078000	-0.038497000
H	2.981535000	1.141997000	1.893249000

(pBrPh)$_3$N

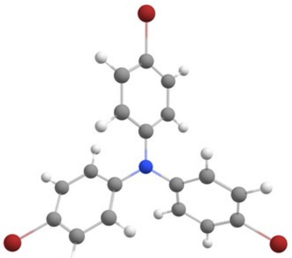

Br	-5.310619000	-2.993915000	-0.000360000
C	-3.660398000	-2.063353000	0.000010000
C	-2.608189000	-2.527031000	0.781860000
C	-1.402427000	-1.837464000	0.788960000
C	-1.232078000	-0.694854000	0.000440000
N	0.000020000	-0.000460000	0.000380000
C	0.014382000	1.413832000	0.000180000
C	-0.888586000	2.132361000	0.790570000
C	-0.883253000	3.521371000	0.783690000
C	0.042636000	4.201107000	-0.000020000
Br	0.061927000	6.095475000	-0.000130000
C	0.954517000	3.502599000	-0.783680000
C	0.931675000	2.113774000	-0.790380000
C	1.217626000	-0.719888000	0.000240000
C	1.364085000	-1.866614000	-0.787150000
C	2.555568000	-2.580530000	-0.780350000
C	3.617685000	-2.137504000	0.000090000
Br	5.248807000	-3.101132000	-0.000140000
C	3.493058000	-0.993486000	0.780610000
C	2.292667000	-0.294653000	0.787530000
C	-2.297611000	-0.248575000	-0.788130000
C	-3.511975000	-0.922884000	-0.781550000
H	-2.732523000	-3.413044000	1.394380000
H	-0.584267000	-2.188906000	1.409180000
H	-1.600654000	1.599255000	1.412160000
H	-1.587188000	4.071825000	1.397780000
H	1.669474000	4.038576000	-1.397860000
H	1.632769000	1.566269000	-1.411930000
H	0.538457000	-2.202032000	-1.406350000
H	2.661287000	-3.469536000	-1.392040000
H	4.322719000	-0.657041000	1.392270000
H	2.187986000	0.590370000	1.406720000
H	-2.174370000	0.633522000	-1.408080000
H	-4.334122000	-0.570300000	-1.394260000

(pBrPh)$_3$N$^{•+}$

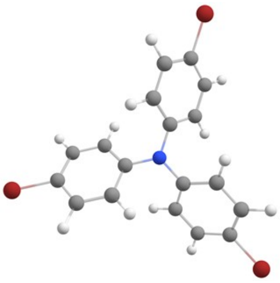

Br	-1.702760000	-5.807438000	-0.000745000
C	-1.176272000	-4.011060000	-0.000042000
C	-0.070644000	-3.615460000	0.758952000
C	0.323236000	-2.289927000	0.758203000
C	-0.395433000	-1.348847000	0.000637000
N	0.000196000	0.000056000	0.000656000
C	-0.970345000	1.017042000	0.000556000
C	-2.144056000	0.865354000	0.759101000
C	-3.095298000	1.869025000	0.760097000
C	-2.885919000	3.023944000	0.000314000
Br	-4.178349000	4.378134000	0.000084000
C	-1.722440000	3.179200000	-0.759389000
C	-0.764143000	2.182268000	-0.758183000
C	1.366133000	0.331973000	0.000401000
C	2.272295000	-0.430513000	-0.756944000
C	3.614832000	-0.099254000	-0.758320000
C	4.062029000	0.987132000	-0.000186000
Br	5.881021000	1.429200000	-0.000486000
C	3.166420000	1.747170000	0.758241000
C	1.821599000	1.425338000	0.757462000
C	-1.508578000	-1.752307000	-0.757080000
C	-1.893104000	-3.080570000	-0.758549000
H	0.466519000	-4.343533000	1.355565000

H	1.162910000	-1.972301000	1.367033000
H	-2.288169000	-0.020362000	1.368494000
H	-3.993891000	1.768028000	1.357511000
H	-1.579645000	4.072003000	-1.356939000
H	0.127327000	2.284783000	-1.367598000
H	1.915367000	-1.254792000	-1.365054000
H	4.316738000	-0.670335000	-1.354789000
H	3.528191000	2.576733000	1.354482000
H	1.126541000	1.993847000	1.365981000
H	-2.043639000	-1.031017000	-1.365428000
H	-2.738410000	-3.402838000	-1.355349000

TCNQ

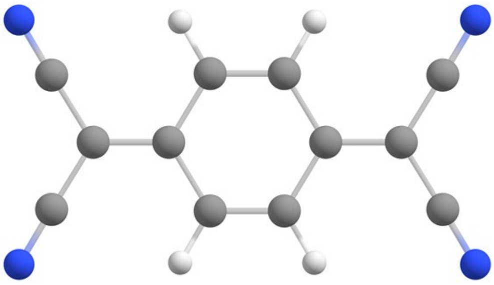

N	-4.142947000	2.214407000	0.000656000
N	4.142849000	-2.214449000	0.000623000
N	4.142947000	2.214407000	0.000656000
N	-4.142850000	-2.214449000	0.000623000
C	3.550703000	-1.217261000	0.000370000
C	-3.550734000	1.217256000	0.000342000
C	-3.550703000	-1.217260000	0.000370000
C	3.550733000	1.217256000	0.000342000
C	-2.797330000	0.000008000	0.000028000
C	2.797330000	0.000008000	0.000028000
C	1.425118000	0.000013000	-0.000486000
C	-1.425118000	0.000013000	-0.000486000
C	-0.673372000	1.245064000	-0.000739000
C	0.673372000	1.245064000	-0.000739000
C	0.673372000	-1.245034000	-0.000711000
C	-0.673372000	-1.245034000	-0.000711000
H	-1.218344000	-2.183924000	-0.000863000
H	1.218344000	-2.183924000	-0.000864000
H	-1.218344000	2.183952000	-0.000912000
H	1.218344000	2.183952000	-0.000912000

TCNQ$^{\bullet-}$

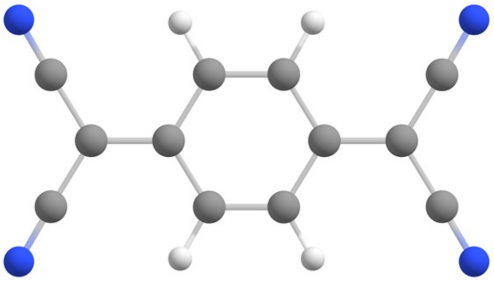

N	-4.182781000	2.214529000	0.000021000
N	4.182814000	-2.214506000	0.000046000
N	4.182782000	2.214529000	0.000017000
N	-4.182814000	-2.214506000	0.000041000
C	3.593456000	-1.208229000	0.000017000
C	-3.593434000	1.208245000	0.000013000
C	-3.593456000	-1.208229000	0.000015000
C	3.593434000	1.208245000	0.000010000
C	-2.852781000	0.000001000	-0.000034000
C	2.852781000	0.000001000	-0.000035000
C	1.431654000	-0.000011000	-0.000040000
C	-1.431654000	-0.000011000	-0.000040000
C	-0.684936000	1.214871000	0.000006000
C	0.684936000	1.214871000	0.000006000
C	0.684935000	-1.214899000	-0.000029000
C	-0.684935000	-1.214899000	-0.000029000
H	-1.218934000	-2.161337000	-0.000040000
H	1.218934000	-2.161337000	-0.000040000
H	-1.218933000	2.161312000	0.000030000
H	1.218933000	2.161312000	0.000029000

TCNQ_(B(C_6F_5)$_3$)$_4$

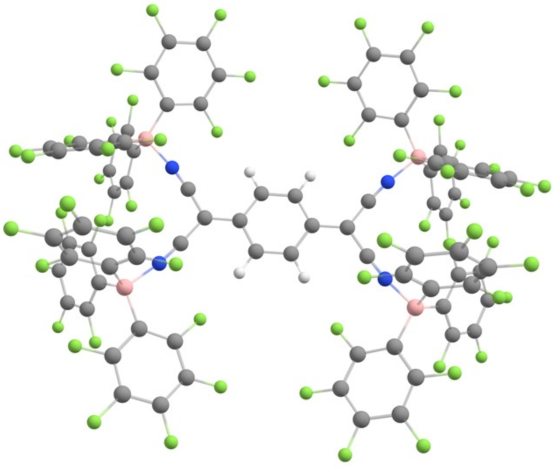

F	5.312373000	-7.136878000	2.957940000
F	5.686208000	-0.246637000	3.842534000
F	8.381359000	0.087135000	3.591680000
F	-6.040157000	2.721902000	5.500629000
F	-8.635072000	3.646950000	-3.984818000
F	-3.539731000	2.706367000	4.414459000
F	-9.669079000	1.180982000	-3.493306000
F	-6.620742000	4.587943000	-2.536757000
F	-8.211409000	2.928731000	3.879477000
F	-7.925800000	3.073445000	1.243658000
F	4.361343000	-1.814741000	2.144293000
F	2.792687000	-7.807194000	2.185091000
F	9.722543000	-1.231978000	1.625746000
F	-2.996504000	8.340546000	-1.059110000
F	-8.640608000	-0.339286000	-1.478753000
F	7.523456000	-1.470988000	-4.325840000
F	6.553668000	-5.045870000	1.857428000
F	-5.486603000	8.036109000	-0.002452000
F	8.503964000	-3.928098000	-5.016655000
F	-1.566699000	6.135646000	-1.795464000
F	1.531173000	-6.328482000	0.267922000
F	6.061639000	-1.180037000	-2.131282000
F	-6.596987000	0.586022000	-0.013346000
F	8.438120000	-2.837156000	-0.046863000
F	-3.220335000	2.886575000	1.766489000
F	-6.524485000	5.583760000	0.316824000
F	7.989820000	-6.072532000	-3.426222000
F	6.570420000	-5.799196000	-1.198055000
F	-2.579614000	3.704017000	-1.505378000
F	2.751195000	-4.246330000	-0.852545000
F	-5.312192000	-7.136866000	2.958163000
F	-5.686062000	-0.246629000	3.842742000
F	-8.381223000	0.087135000	3.591986000
F	6.040285000	2.721892000	5.500440000
F	8.634943000	3.647016000	-3.985074000
F	3.539832000	2.706350000	4.414333000

F	9.668978000	1.181049000	-3.493618000
F	6.620650000	4.587981000	-2.536947000
F	8.211495000	2.928730000	3.879233000
F	7.925819000	3.073445000	1.243422000
F	-4.361257000	-1.814737000	2.144459000
F	-2.792547000	-7.807182000	2.185180000
F	-9.722477000	-1.231988000	1.626107000
F	2.996433000	8.340546000	-1.059171000
F	8.640573000	-0.339247000	-1.479053000
F	-7.523603000	-1.471034000	-4.325575000
F	-6.553550000	-5.045864000	1.857711000
F	5.486564000	8.036115000	-0.002586000
F	-8.504142000	-3.928152000	-5.016323000
F	1.566618000	6.135644000	-1.795500000
F	-1.531139000	-6.328475000	0.267938000
F	-6.061702000	-1.180059000	-2.131076000
F	6.596990000	0.586035000	-0.013575000
F	-8.438112000	-2.837171000	-0.046543000
F	3.220368000	2.886561000	1.766372000
F	6.524467000	5.583768000	0.316641000
F	-7.989943000	-6.072566000	-3.425882000
F	-6.570460000	-5.799204000	-1.197769000
F	2.579555000	3.704018000	-1.505462000
F	-2.751224000	-4.246328000	-0.852470000
N	-4.235006000	1.956796000	-0.510384000
N	4.304006000	-2.196405000	-0.488778000
N	4.234987000	1.956797000	-0.510532000
N	-4.304018000	-2.196403000	-0.488616000
C	6.356581000	-0.874081000	2.875662000
C	7.728465000	-0.706304000	2.752362000
C	8.407906000	-1.378387000	1.746782000
C	-5.884833000	2.807219000	4.186828000
C	-4.612440000	2.801056000	3.632126000
C	-8.672385000	1.645157000	-2.752749000
C	5.697610000	-1.703692000	1.982446000
C	-8.141048000	2.902983000	-2.999411000
C	-6.991650000	2.902606000	3.355328000
C	3.401677000	-6.762667000	1.639230000
C	5.308618000	-5.314800000	1.456479000
C	-6.814984000	2.978638000	1.978234000
C	4.687351000	-6.414511000	2.034410000
C	-3.509227000	7.127272000	-0.899134000
C	-4.780242000	6.968545000	-0.358327000
C	-8.146969000	0.872760000	-1.725138000
C	7.511243000	-4.875180000	-3.102213000
C	2.762009000	-6.011097000	0.663842000
C	7.276895000	-2.536266000	-3.566364000
C	7.708765000	-2.204349000	0.873691000
C	7.777954000	-3.782728000	-3.915980000
C	-4.480475000	2.890170000	2.255022000
C	6.328242000	-2.376958000	0.938276000
C	-7.086835000	3.375839000	-2.222042000
C	-5.294313000	5.690850000	-0.196805000
C	-2.784574000	6.006070000	-1.273924000
C	-7.098306000	1.380186000	-0.979798000
C	6.516982000	-2.419204000	-2.414621000
C	-6.533243000	2.637788000	-1.180699000
C	-5.558642000	2.970137000	1.377542000
C	4.700662000	-4.523371000	0.485253000
C	3.425624000	-4.926202000	0.108357000
C	6.753310000	-4.708202000	-1.948029000
C	-4.603948000	4.535237000	-0.555647000
C	-3.345584000	4.746899000	-1.099273000
C	3.565801000	-1.327386000	-0.598406000
C	-3.560987000	1.034595000	-0.604937000
C	6.229414000	-3.478924000	-1.558915000
C	-6.356470000	-0.874079000	2.875898000
C	-7.728358000	-0.706305000	2.752647000
C	-8.407835000	-1.378395000	1.747096000
C	5.884928000	2.807210000	4.186644000
C	4.612521000	2.801044000	3.631974000
C	8.672303000	1.645210000	-2.753028000
C	-5.697529000	-1.703692000	1.982660000
C	8.140952000	2.903036000	-2.999661000
C	6.991723000	2.902601000	3.355116000
C	-3.401568000	-6.762658000	1.639349000
C	-5.308522000	-5.314794000	1.456696000
C	6.815022000	2.978635000	1.978026000
C	-4.687221000	-6.414502000	2.034596000
C	3.509166000	7.127274000	-0.899219000
C	4.780198000	6.968550000	-0.358449000
C	8.146920000	0.872799000	-1.725411000
C	-7.511351000	-4.875211000	-3.101906000
C	-2.761954000	-6.011090000	0.663923000
C	-7.277015000	-2.536303000	-3.566095000
C	-7.708725000	-2.204359000	0.873982000
C	-7.778090000	-3.782769000	-3.915677000
C	4.480521000	2.890160000	2.254873000
C	-6.328199000	-2.376964000	0.938516000
C	7.086759000	3.375877000	-2.222257000
C	5.294280000	5.690856000	-0.196952000
C	2.784509000	6.006071000	-1.273996000
C	7.098277000	1.380212000	-0.980034000
C	-6.517059000	-2.419229000	-2.414382000
C	6.533201000	2.637813000	-1.180905000
C	5.558665000	2.970132000	1.377366000
C	-4.700620000	-4.523369000	0.485432000
C	-3.425601000	-4.926199000	0.108469000
C	-6.753375000	-4.708220000	-1.947753000
C	4.603911000	4.535242000	-0.555784000
C	3.345530000	4.746901000	-1.099372000
C	-3.565818000	-1.327384000	-0.598272000
C	3.560969000	1.034593000	-0.605066000
C	-6.229460000	-3.478939000	-1.558675000
C	-2.777984000	-0.149077000	-0.665316000
C	2.777964000	-0.149079000	-0.665420000
C	1.405932000	-0.147784000	-0.695875000
C	-1.405953000	-0.147784000	-0.695824000
C	-0.672851000	1.106429000	-0.705589000
C	0.672830000	1.106429000	-0.705613000
C	0.672860000	-1.403322000	-0.701281000
C	-0.672881000	-1.403321000	-0.701256000
B	-5.302310000	3.109759000	-0.228551000
B	5.464098000	-3.239465000	-0.146782000
B	5.302291000	3.109765000	-0.228719000
B	-5.464091000	-3.239469000	-0.146573000
H	-1.221506000	-2.341705000	-0.697500000
H	1.221484000	-2.341705000	-0.697544000
H	-1.220092000	2.044838000	-0.712206000
H	1.220071000	2.044838000	-0.712250000

TCNQ_$(B(C_6F_5)_3)_4^{\bullet -}$

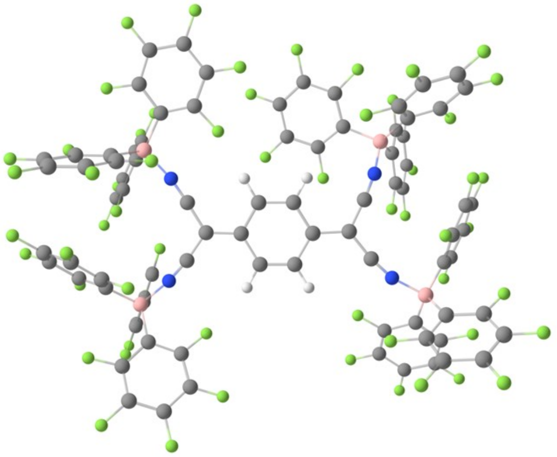

F	9.591272000	3.768555000	-4.090738000
F	7.912844000	-1.695427000	-1.031705000
F	9.002950000	-1.781115000	1.479176000
F	-8.560304000	-1.791944000	-4.769867000
F	-6.026159000	-3.906020000	4.530390000
F	-5.855118000	-1.745058000	-5.103786000
F	-7.200347000	-1.477679000	4.879734000
F	-4.995562000	-4.591619000	2.182991000
F	-9.602957000	-2.374860000	-2.324691000
F	-8.018710000	-2.841393000	-0.253458000
F	6.664388000	0.495896000	-1.871934000
F	7.767806000	5.163639000	-5.548515000
F	8.808876000	0.389345000	3.093792000
F	-2.830133000	-8.083691000	-1.396827000
F	-7.336456000	0.269532000	2.788576000
F	3.513860000	6.140563000	2.401795000
F	8.925137000	2.707088000	-1.744583000
F	-5.498429000	-7.703040000	-1.001468000
F	5.739874000	7.623072000	2.922620000
F	-1.127847000	-5.958881000	-1.213604000
F	5.233861000	5.484272000	-4.594702000
F	3.658217000	3.956889000	0.903857000
F	-6.280663000	-0.388507000	0.422678000
F	7.545187000	2.573750000	2.295504000
F	-4.234529000	-2.258028000	-3.044851000
F	-6.443299000	-5.261235000	-0.446970000
F	8.142070000	6.854590000	1.892898000
F	8.311546000	4.647851000	0.377574000
F	-2.030649000	-3.551029000	-0.600977000
F	4.543746000	4.430336000	-2.229474000
F	-6.216836000	7.495755000	-2.559155000
F	-7.112199000	1.024488000	-3.793550000
F	-9.462369000	0.461298000	-2.533390000
F	7.549779000	-6.053854000	-3.732577000
F	6.318652000	-4.789740000	4.598169000
F	4.972062000	-5.292481000	-4.192837000
F	6.771471000	-2.268056000	5.526910000
F	5.280585000	-5.157567000	2.156964000
F	8.717145000	-5.478721000	-1.342853000
F	7.382417000	-4.188769000	0.541908000
F	-5.211414000	2.413987000	-2.534840000
F	-3.634433000	8.331254000	-2.381443000
F	-9.861383000	1.322336000	0.018050000
F	-0.805713000	-5.309374000	1.490869000
F	6.146702000	-0.125875000	3.953249000
F	-5.214246000	1.419276000	4.785404000
F	-6.996693000	5.244430000	-1.370454000
F	1.098452000	-6.972383000	0.479211000
F	-6.105858000	3.746611000	5.906603000
F	-0.208179000	-2.682552000	1.914505000
F	-1.829530000	6.846791000	-0.972442000
F	-4.781381000	1.260796000	2.182099000
F	5.154430000	-0.459945000	1.542324000
F	-8.007925000	2.729045000	1.278415000
F	3.595476000	-3.997307000	-2.296777000
F	3.523160000	-6.068886000	-0.091765000
F	-6.571995000	5.906248000	4.317032000
F	-6.191156000	5.762553000	1.692359000
F	2.240235000	-1.767320000	1.441182000
F	-2.586925000	4.588429000	0.229040000
N	-3.911969000	-1.695827000	-0.439959000
N	4.830085000	2.219170000	-0.677560000
N	4.153895000	-1.891304000	-0.480892000
N	-4.060266000	2.407435000	-0.071230000
C	7.841685000	-0.635278000	-0.225474000
C	8.392696000	-0.683190000	1.047745000
C	8.290435000	0.429089000	1.867705000
C	-7.755420000	-2.035583000	-3.740940000
C	-6.378616000	-2.012947000	-3.907973000
C	-6.694346000	-1.810424000	3.696378000
C	7.207626000	0.524013000	-0.639232000
C	-6.096132000	-3.047824000	3.512442000
C	-8.282940000	-2.327036000	-2.491967000
C	7.422518000	4.634483000	-4.376807000
C	7.974726000	3.371795000	-2.409097000
C	-7.428955000	-2.567995000	-1.422164000
C	8.349203000	3.921018000	-3.630798000
C	-3.297603000	-6.870570000	-1.116328000
C	-4.658489000	-6.671764000	-0.917755000
C	-6.760342000	-0.921895000	2.631968000
C	7.048084000	6.135361000	1.641927000
C	6.132695000	4.795924000	-3.889790000
C	4.694484000	5.772206000	1.903673000
C	7.629548000	1.569296000	1.417775000
C	5.824345000	6.529176000	2.168971000
C	-5.561463000	-2.273717000	-2.816989000
C	7.075086000	1.669089000	0.146209000
C	-5.566727000	-3.384590000	2.269235000
C	-5.124635000	-5.396788000	-0.637189000
C	-2.435914000	-5.791099000	-1.018042000
C	-6.214756000	-1.294341000	1.415963000
C	4.802676000	4.631414000	1.113979000
C	-5.612812000	-2.527987000	1.175347000
C	-6.041907000	-2.539330000	-1.537352000
C	6.692182000	3.500486000	-1.886840000
C	5.801447000	4.234685000	-2.664615000
C	7.109608000	4.994189000	0.858243000
C	-4.298781000	-4.278954000	-0.532973000
C	-2.946990000	-4.534535000	-0.714502000
C	3.984686000	1.453249000	-0.839841000
C	-3.257305000	-0.750568000	-0.503814000
C	6.004180000	4.198459000	0.567871000
C	-7.314930000	1.431236000	-2.538699000
C	-8.512621000	1.143771000	-1.899575000
C	-8.712889000	1.587340000	-0.601976000
C	6.862977000	-5.414852000	-2.788172000
C	5.549497000	-5.027316000	-3.020174000
C	6.263425000	-2.451563000	4.311738000

C	-6.348047000	2.157750000	-1.861491000
C	6.030670000	-3.735898000	3.835083000
C	7.452307000	-5.123699000	-1.568811000
C	-4.012439000	7.200260000	-1.791107000
C	-5.707848000	5.584349000	-1.262577000
C	6.723778000	-4.451981000	-0.591166000
C	-5.328367000	6.768554000	-1.882252000
C	0.406823000	-4.845725000	1.203328000
C	1.380262000	-5.689061000	0.688912000
C	5.950293000	-1.367378000	3.509632000
C	-6.121214000	4.770824000	3.782809000
C	-3.096522000	6.444381000	-1.074292000
C	-5.430005000	2.493231000	4.026873000
C	-7.717442000	2.316553000	0.040937000
C	-5.887380000	3.671537000	4.596483000
C	4.861034000	-4.362284000	-2.019107000
C	-6.495589000	2.616240000	-0.553446000
C	5.493978000	-3.900447000	2.568484000
C	2.645993000	-5.188630000	0.398798000
C	0.715445000	-3.512644000	1.427219000
C	5.424340000	-1.580413000	2.239002000
C	-5.208594000	2.444986000	2.658399000
C	5.185607000	-2.844054000	1.712993000
C	5.402725000	-4.059346000	-0.771212000
C	-4.822134000	4.786797000	-0.544638000
C	-3.520640000	5.270102000	-0.466937000
C	-5.895575000	4.674058000	2.415315000
C	3.003884000	-3.857548000	0.601238000
C	1.999633000	-3.062092000	1.153102000
C	-3.307620000	1.566642000	-0.297793000
C	3.721696000	-0.863580000	-0.772257000
C	-5.425486000	3.517885000	1.798688000
C	-2.516148000	0.440500000	-0.553717000
C	3.105919000	0.374714000	-0.987962000
C	1.684873000	0.444726000	-1.022485000
C	-1.115810000	0.467207000	-0.778482000
C	-0.422352000	-0.748657000	-1.031878000
C	0.933900000	-0.757272000	-1.153116000
C	0.986809000	1.672425000	-0.852337000
C	-0.376007000	1.683183000	-0.728962000
B	-4.999584000	-2.828037000	-0.307195000
B	6.211854000	2.935466000	-0.432526000
B	4.489921000	-3.223260000	0.286037000
B	-5.259004000	3.395279000	0.181950000
H	-0.894884000	2.621869000	-0.554596000
H	1.545527000	2.600077000	-0.765510000
H	-0.973404000	-1.678862000	-1.115071000
H	1.445450000	-1.697491000	-1.335635000

VI. References

[1] Wang, J. and Yang, S. *Tetrahedron Lett.* **2016**, *57* (31), 3444-3448.
[2] Armarego, W. L. G.; Chai, C. C. L. Purification of Laboratory Chemicals. 6th ed. Oxford: Butterworth-Heinemann; 2009. p. 180 & 558.
[3] Lawrence, E. J.; Oganesyan, V. S.; Wildgoose, G. G.; Ashley, A. E. *Dalton Trans.* **2013**, *42*, 782-789.
[4] Elgrishi, N.; Rountree, K. J.; McCarthy, B. D.; Rountree, E. S.; Eisenhart, T. T.; Dempsey, J. L. *J. Chem. Educ.* **2018**, *95* (2), 197-206.
[5] Espinoza, E. M.; Clark, J. A.; Soliman, J.; Derr, J. B.; Morales, M.; Vullev, V. I. *J. Electrochem. Soc.* **2019**, *166* (5), H3175-H3187.
[6] Zagumennov, V. A.; Nikitin, E. V. *Russ. J. Electrochem.***2003**, *39* (11), 1236-1239.
[7] Tao, X.; Daniliuc, C. G.; Janka, O.; Pöttgen, R.; Knitsch, R.; Hansen, M. R.; Eckert, H.; Lübbesmeyer, M.; Studer, A.; Kehr, G.; Erker, G. *Angew. Chem. Int. Ed.* **2017**, *56* (52), 16641-16644. *Angew. Chem.* **2017**, *129* (52), 16868-16871.
[8] Connelly, N. G.; Geiger, W. E. *Chem. Rev.* **1996**, *96* (2), 877-910.
[9] Lu, Z.; Ju, M.; Wang, Y.; Meinhardt, J. M.; Martinez Alvarado, J. I.; Villemure, E.; Terrett, J. A.; Lin, S. *Nat.* **2023**, *619*, 514-520.
[10] Medina Rivero, A.; Urieta-Mora, J.; Molina-Ontoria, A.; Martín-Fuentes, C.; Urgel, J. I.; Zubiria-Ulacia, M.; Lloveras, V.; Casanova, D.; Martínez, J. I.; Veciana, J.; Écija, D.; Martín, N.; Casado, J. *Angew. Chem. Int. Ed.* **2021**, *60* (33), 17887-17892. *Angew. Chem.* **2021**, *133* (33), 18031-18036.
[11] Albrecht, P. A.; Rupf, S. M.; Sellin, M.; Schlögel, J.; Riedel, S.; Malischewski, M. *Chem. Commun.* **2022**, *58*, 4958-4961.
[12] Barham, J.; John, M. P.; Murphy, J. A. *J. Am. Chem. Soc.* **2016**, *138* (47), 15842-15847.
[13] Tajima, T.; Fuchigami, T. *Angew. Chem. Int. Ed.* **2005**, *44* (30), 4760-4763. *Angew. Chem.* **2005**, *117* (30), 4838-4841.
[14] Mizuta, S.; Verhoog, S.; Wang, X.; Shibata, N.; Gouverneur, V.; Médebielle, M. *J. Fluor. Chem.* **2013**, *155*, 124-131.
[15] Holtrop, F.; Jupp, A. R.; van Leest, N. P.; Paradiz Dominguez, M.; Williams, R. M.; Brouwer, A. M.; de Bruin, B.; Ehlers, A. W.; Slootweg, J. C. *Chem. Eur. J.* **2020**, *26* (41), 9005-9011.
[16] Merk, A. Großekappenber, H.; Schmidtmann, M.; Leucke, M. P.; Lorent, C.; Driess, M.; Oestreich, M.; Klare, H. F. T.; Müller, T. *Angew. Chem. Int. Ed.* **2018**, *57* (46), 15267-15271. *Angew. Chem.* **2018**, *130* (46), 15487-15492.
[17] Cheng, Y.; Yuan, X.; Ma, J.; Yu, S. *Chem. Eur. J.* **2015**, *21* (23), 8355-8359.
[18] Stoll, S.; Schweiger, A. *J. Magn. Reson.* **2006**, *178* (1), 42-55.
[19] Chai, J. D.; Head-Gordon, M. *Phys. Chem. Chem. Phys*. **2008**, *10*, 6615–6620.
[20] Blaudeau, J. P.; McGrath, M. P.; Curtiss, L. A.; Radom, L. *J. Chem. Phys*. **1997**, *107* (13), 5016-5021.
[21] M. M. Francl, M. M; Pietro, W. J.; Hehre, W. J.; Binkley, J. S.; DeFrees, D. J.; Pople, J. A.; Gordon, M. S. *J. Chem. Phys*. **1982**, *77*, 3654–3665.
[22] Gaussian 16, Revision C.01, Frisch, M. J.; Trucks, G. W.; Schlegel, H. B.; Scuseria, G. E.; Robb, M. A.; Cheeseman, J. R.; Scalmani, G.; Barone, V.; Petersson, G. A.; Nakatsuji, H.; Li, X.; Caricato, M.; Marenich, A. V.; Bloino, J.; Janesko, B. G.; Gomperts, R.; Mennucci, B.; Hratchian, H. P.; Ortiz, J. V.; Izmaylov, A. F.; Sonnenberg, J. L.; Williams-Young, D.; Ding, F.; Lipparini, F.; Egidi, F.; Goings, J.; Peng, B.; Petrone, A.; Henderson, T.; Ranasinghe, D.; Zakrzewski, V. G.; Gao, J.; Rega, N.; Zheng, G.; Liang, W.; Hada, M.; Ehara, M.; Toyota, K.; Fukuda, R.; Hasegawa, J.; Ishida, M.; Nakajima, T.; Honda, Y.; Kitao, O.; Nakai, H.; Vreven, T.; Throssell, K.; Montgomery, Jr., J. A.; Peralta, J. E.; Ogliaro, F.; Bearpark, M. J.; Heyd, J.

J.; Brothers, E. N.; Kudin, K. N.; Staroverov, V. N.; Keith, T. A.; Kobayashi, R.; Normand, J.; Raghavachari, K.; Rendell, A. P.; Burant, J. C.; Iyengar, S. S.; Tomasi, J.; Cossi, M.; Millam, J. M.; Klene, M.; Adamo, C.; Cammi, R.; Ochterski, J. W.; Martin, R. L.; Morokuma, K.; Farkas, O.; Foresman, J. B.; Fox, D. J.; Gaussian, Inc., Wallingford CT, 2016.
[23] Scalmani, G.; Frisch, M. J. *J. Chem. Phys*. **2010**, *132*, 114110.
